# Mechanistic Pathways of Wound Healing: From Hemostasis to Scar‐Free Regeneration

**DOI:** 10.1002/cbf.70309

**Published:** 2026-09-18

**Authors:** Devadass Jessy Mercy, Koyeli Girigoswami, Agnishwar Girigoswami

**Affiliations:** ^1^ Medical Bionanotechnology, Faculty of Allied and Healthcare Sciences, Chettinad Hospital & Research Institute, Chettinad Academy of Research and Education, Kelambakkam Chennai Tamil Nadu India; ^2^ Medical Bionanotechnology Lab, Department of Obstetrics and Gynecology Saveetha Medical College and Hospital, Saveetha Institute of Medical and Technical Sciences, Thandalam Chennai Tamil Nadu India

**Keywords:** cellular mechanism, chronic wounds, extracellular matrix, health care, therapy

## Abstract

Wound healing is the critical process regulated through a harmonious coordination of cellular, molecular, pathological, and systemic communications. Normally, healing occurs in four overlapping phases, which are modified and controlled by various growth factor interactions and signaling pathways. Disruption of the tightly coordinated wound‐healing cascade, together with aberrant protein signaling, mitochondrial dysfunction, antimicrobial resistance, sustained fibroblast activation, and maladaptive epigenetic reprogramming, drives pathological tissue repair culminating in chronic non‐healing wounds, including diabetic, ischemic, and venous ulcers, as well as fibrotic outcomes such as hypertrophic and keloid scarring. The persistent fibroblast activation, reduced apoptosis, prolonged TGF‐β activity, and severe fibrosis induced by mechanical methods trigger excessive healing, while existing therapies fail to manage these functions. Therefore, in order to develop advancements toward precise and personalized medicine, it is important to understand the various mechanisms involved in wound healing. The current review provides a comprehensive understanding of various fundamental mechanisms involved in wound healing, such as cellular, molecular, signaling pathways, genetic, epigenetic, pathological, immunological, and systemic mechanisms. Knowledge of these mechanisms and how they work under physiological as well as pathological conditions connects to clinical inefficiency, which helps to provide accurate medicine. A deeper mechanistic knowledge paves the way for developing innovative biomaterials; nanotechnology‐based therapeutic strategies, specific drug delivery, molecular biomarkers, and scar‐free healing of wounds, which enrich the future with advanced therapeutics and personalized medicine for wounds.

## Significance

Wound healing is a tightly coordinated biological process involving overlapping cellular, molecular, and systemic events, yet current knowledge is often fragmented across disciplines. This review integrates cellular participants, extracellular matrix dynamics, immune responses, genetic and epigenetic regulation, and key signaling pathways, including TGF‐β/SMAD, NF‐κB, MAPK, PI3K/Akt, and Wnt/β‐catenin, within the four classical phases of healing. By linking mechanistic regulation to pathological outcomes such as chronic wounds, diabetic and ischemic ulcers, and fibrotic scarring, it clarifies how dysregulated cell signaling and tissue responses impair repair. It also highlights the limitations of symptom‐based treatments and emphasizes mechanism‐guided strategies, including nanotechnology and regenerative approaches, to improve healing quality. This integrated perspective connects cell biochemistry with clinical practice and supports the development of targeted, next‐generation therapies.

## 1 Introduction

Wound healing is a critically dynamic and biologically synchronized process that specifically restores the integrity of the wound site. This synchronized process includes various cellular key players, like immune cells, keratinocytes, fibroblasts, and endothelial cells, which bridge the crucial connection between molecular pathways, involving cytokines, chemokines, growth factors, cellular signaling, and mechanical stability from the extracellular matrix (ECM) [1]. As wound healing involves four essential phases, like hemostasis, inflammation, proliferation, and remodeling, any alteration or abnormality in any of these phases results in delayed wound closure, dysregulated tissue remodeling, and impaired wound healing. Wounds have now become a worldwide issue, excluding the fact that it is a “clinical concern” due to the increase in cases of diabetes, blood diseases, trauma, an aging population, and invasive operating procedures, which make it a major drawback among the public [2]. Hence, the deeper insight into the wound healing mechanisms provides essential knowledge and enables researchers to develop novel strategies to achieve scar‐free tissue regeneration.

### 1.1 Clinical Significance of Wound Healing

Healing of wounds is essential because the skin, the outermost layer, maintains the body and other parts and protects them from external pathogens, fluid loss, and infections [3]. Beyond its physiological role, wound healing is crucial for stability, function, and mental well‐being. So, when the healing process is disrupted, a normal wound can progress into a non‐healing or chronic wound, leading to sustained hospitalization, increased risk of infections, sepsis, and even amputation in cases of diabetic or ischemic conditions [4]. However, in the case of burn injury, an early and rapid wound healing process is required to minimize the mortality rate, and similarly, surgical interventions delay the wound healing process, which increases the revisit to the hospital, causing severe trauma or mental distress to the affected person [5]. At the molecular level, wound healing displays how the body carefully coordinates cell signaling, new blood vessel growth, tissue remodeling, and immune regulation in a step‐by‐step sequence. So, the imbalance of wounds in diabetic or ischemic individuals and excessive healing in hypertrophic or keloid scars differentiates the various pathological states. Hence, complete wound healing does not depend only on the closure of an open wound; it depends on how mechanisms like metabolism, blood vessel growth, immune response, and tissue movement function. Recognizing both the biological and clinical factors shows that wound healing should be observed through a mechanistic perspective, rather than being viewed as a simple, one‐step tissue repair event [6].

### 1.2 Universal Burden of Chronic Wounds and Scars

About 5%–10% of the worldwide population, especially older adults and diabetic patients, suffer from chronic wounds involving diabetic ulcers, ischemic ulcers, venous leg ulcers, and pressure ulcers. In the United States, chronic wound care costs over $30 billion annually, with Europe and developing nations facing equivalent burdens [7]. However, amputation rates have increased in the case of diabetic ulcers, whereas repeated ulcer formation reduces the need and value of life, causing both physical and psychological issues for patients worldwide. Likewise, hypertrophic scars and keloid scars indicate abnormal fibrotic issues, which do not heal rapidly. Moreover, these scars are commonly observed in adults with darker skin, even causing pain, itching, and restricted movement, affecting healing and causing mental stress. Despite the development of several treatments presented, the recurrence of keloid scars often increases by about 50%–90%, indicating its critical mechanism and pathogenesis. Hence, both chronic wounds and fibrotic scars together form a mechanism that specifically affects the healing process, ensuring the universal need for mechanism‐based therapeutic approaches [8].

### 1.3 Limitations of Current Therapies

Traditional wound healing treatments like surgical grafts, dressings, antibiotics, and anti‐inflammatory drugs specifically relieve the symptoms but do not assist with the deeper complications. Unfortunately, most chronic wounds fail to completely heal due to poor vascular growth, sustained inflammation, biofilm formation, and metabolic impairment [9]. Ultimately, in severe diabetic conditions, the high sugar level disrupts vascular growth, ages fibroblasts, damages mitochondria, and unbalances the immune system. Following that, in ischemic wounds, the healing process is discontinued due to the shortage of vascular growth. On the contrary, numerous treatments have not been developed to address the molecular characteristics of a wound. Because two wounds can look similar on the surface but differ greatly, which can be analyzed by the behavior of the immune cells, cytokine activity, fibroblast function, and ECM deposition, yet both receive similar therapies [10]. The process of mismatching initiates the critical situation for effective care, and the drawbacks of the present clinical approaches arise from the absence of mechanism‐based classification, reliable biomarkers, and less focus on signaling pathways. So, in order to move away from this symptomatic wound management, understanding the mechanisms at different levels is essential to enhance and enrich the wound healing process.

### 1.4 Rationale for a Broad Mechanistic Review

Wound healing is not considered a biological replacement of tissue, but rather a connecting network of various cellular signaling pathways, immunological responses, metabolic feedback, pathological interventions, and systemic impacts [11]. Recent progress in molecular biology, biomaterials, bioinformatics, tissue engineering, regenerative medicine, and single‐cell transcriptomics displays the highly synchronized interaction among gene expression, epigenetic modifications, and control of blood vessel growth in the neurotic region. However, the knowledge on these is scattered across different studies instead of one. In order to advance this, a comprehensive review comprising all the essential and crucial mechanisms involved in wound healing, which assesses the cellular pathways, molecular pathways, and even Notch signaling involving inflammation, vascular growth, ECM remodeling, immune regulation, and so forth [12].

## 2 Overview of Wound Healing

Wound healing is a fundamental process that involves many factors that help restore the essential healing properties of the skin. It also includes the orderly sequence of different phases of healing, which enhances scar‐free healing. The overview comprises different wound healing phases, such as hemostasis, inflammation, proliferation, and remodeling. Hemostasis is the direct biological response to wounded tissue, initiated by vasoconstriction and platelet adhesion to exposed collagen [13]. Early repair processes are stimulated by immune cells that are recruited through critical mediators such as transforming growth factor‐β (TGF‐β), platelet‐derived growth factor (PDGF), vascular endothelial growth factor (VEGF), and EGF, which are released by platelets. The fibrin clot helps in sealing the wound and acts as a temporary ECM scaffold. It also directs early cell migration through the biochemical signaling center. The activation of key signaling pathways such as TGF‐β/SMAD, PI3K/Akt, and YAP/TAZ mechanotransduction in this stage promotes the activation of fibroblasts and the survival of keratinocytes. Hence, hemostasis is a response that regulates the trajectory of healing paracrine signaling and mechanobiology. Both healthy and pathological healing can become critical if there is a failure in this phase, which might delay immune recruitment or trigger excessive fibrosis [14].

**Figure 1:**
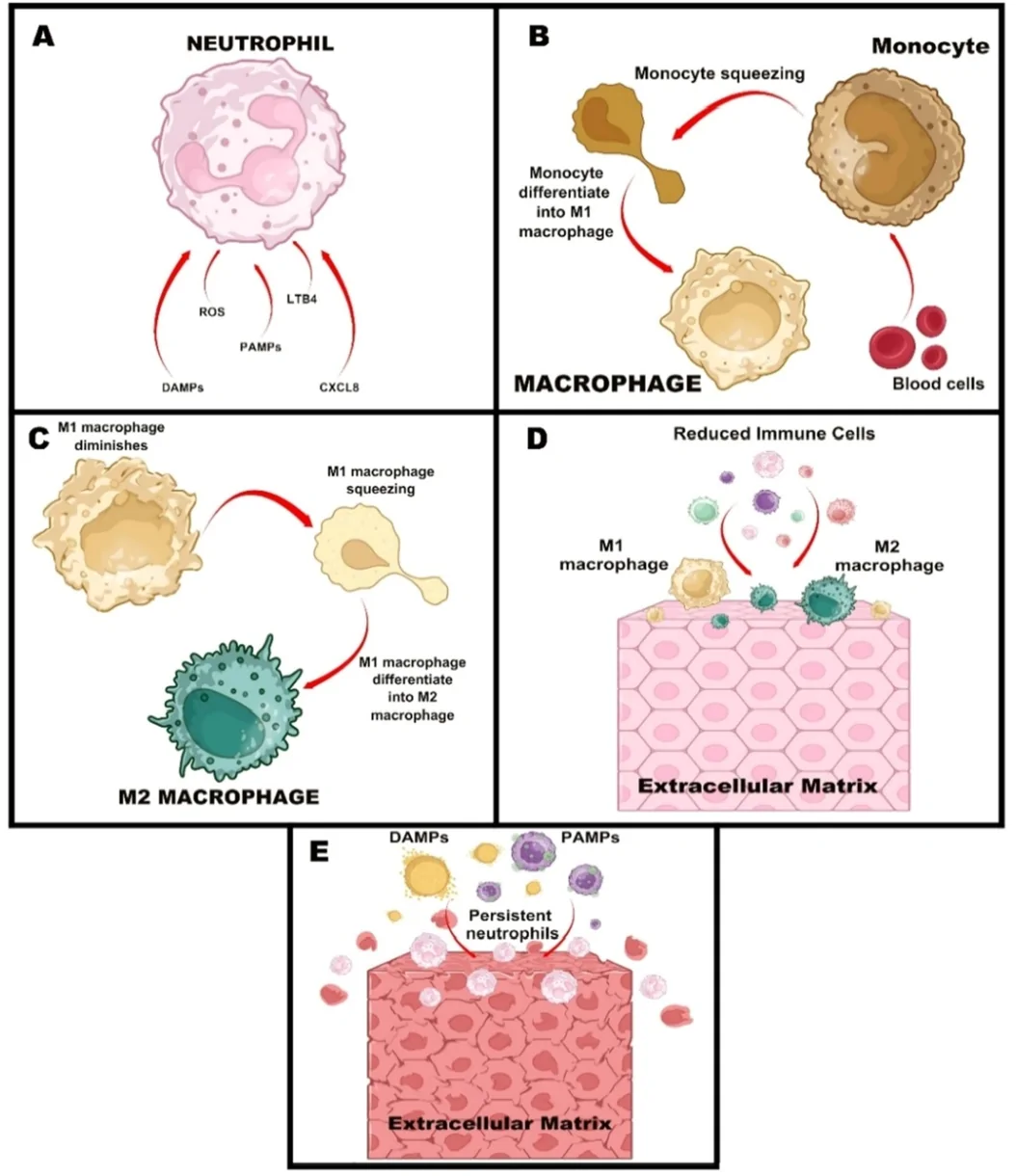
Showing the role of immune cells and their action in wound healing: (A) early inflammation; (B) M1 macrophage activation; (C) restoration of M2 macrophages; (D) tissue regeneration; and (E) chronic inflammation.

The pathogens and debris are eliminated through neutrophils and macrophages in the inflammatory phase. Phagocytosis, ROS generation, and neutrophil extracellular trap (NET) release are used by neutrophils to control infections, but excessive presence can damage fibroblast function and the ECM. Meanwhile, macrophages act as central regulators that move from an M1 inflammatory phenotype to an M2 reparative phenotype. This releases interleukin‐10 (IL‐10), TGF‐β, VEGF, and PDGF that promote regeneration. Cytokine release and immune polarization are controlled by important pathways such as NF‐κB, MAPK, and JAK/STAT [15]. Table 1 describes the role of neutrophils and macrophages in the wound healing process. Dysregulated inflammation leads to M1 activation and chronic non‐healing ulcers. Figure 1A–E describes the role of immune cells and their action in wound healing. These are commonly found in diabetic or ischemic wounds. To prevent fibrosis and ensure a successful transition to the proliferative phase, a timely shift to the M2 phenotype is necessary [21].

**Table 1:** Comparative role of neutrophils and macrophages in wound healing.

Wound healing stages	Time period	Cell entry and action	Molecules released/signals secreted	Shift/outcome	Functions at the wound site	Reference
Hemostasis and early inflammation	0–24 h	**Action:** Undergo extravasation and migrate to the wound (Figure 1A)	ROS, myeloperoxidase, LTB4, CXCL8, elastase and gasdermin	After the clearance of microorganisms, most cells undergo apoptosis, and some may be reversed and migrate to the bloodstream.	Creates NETs to trap microbes. Engulfs the microbes and clears the debris. Releases ROS Forms more neutrophils with packed behavior.	[16]
Late Inflammatory/early proliferative	1–3 days	**Action:** Gets activated by these factors (Figure 1B)	TNF‐α, IL‐6, IL‐1β, LTB4, prostaglandins, MCP‐1	Subsequently, it triggers the transition of monocytes → M1 macrophages following efferocytosis and IL‐4/IL‐13 signaling.	Amplifies inflammation Removes dead cells and other debris. Phagocytize apoptotic neutrophils.	[17]
Proliferative	3–10	**Action:** Stimulated by IL‐4, IL‐3, Treg cytokines (Figure 1C)	VEGFA, PDGF, TGF‐β, FGF2, lipoxins	Implement pro‐resolving functions, minimize inflammation, and promote tissue regeneration.	VEGFA promotes angiogenesis. Stimulates fibroblast division and ECM deposition. Controls keratinocyte movement and regeneration Organizes granulation and tissue formation.	[18]
Remodeling		**Action:** Both macrophages remain active in the ECM matrix (Figure 1D)	TGF‐β, RELMα, Wnt, lipoxins	Macrophages undergo cell death or relocate to lymph nodes, leaving behind mature scar tissue.	Remodels collagen network and ECM deposition. Controls the myofibroblast segregation. Stimulates wound tightening and closure. Resolves inflammation.	[19]
Chronic/pathological wound		**Action:** Transition of M1 → M2 is delayed (Figure 1E)	TNF‐α, IL‐6, prolonged ROS, PAD4	Leads to abnormal or non‐healing wounds.	Increased NETs damage tissues. Prolonged inflammation affects the natural process of angiogenesis and regeneration Delayed ECM remodeling.	[20]

In the proliferation phase, the fibroblasts, driven by the TGF‐β/SMAD, Wnt/β‐catenin, Rho/ROCK, and YAP/TAZ signals, migrate to the wound, proliferate, and differentiate into α‐SMA‐expressing myofibroblasts. Healing of these cells is supported by the secretion of type III collagen, fibronectin, and hyaluronic acid, forming granulation tissue. Likewise, the delivery of nutrients and oxygen to the wound bed is supported by angiogenesis, which helps restore the blood supply through VEGF, hypoxia‐inducible factor‐1α (HIF‐1α), and Notch pathways [22]. As keratinocytes migrate and proliferate under EGF, HGF, and integrin indications, re‐epithelialization occurs. Efficient immune–fibroblast–endothelial coordination is the main factor for this phase. Any disruption to this causes chronic wounds or excessive scarring. Therefore, for a smooth transition, directing towards tissue remodeling is done with the help of effective resolution of inflammation and proper ECM deposition [23].

After re‐epithelialization, type 1 collagen is gradually replaced by immature type II collagen to increase tensile strength, after which remodeling begins, which is strictly regulated by the balance between tissue inhibitors of metalloproteinases (TIMPs) and matrix metalloproteinases (MMPs). As the mechanical tension decreases, myofibroblasts undergo apoptosis, which makes the wound become less cellular and more structured [24]. However, hypertrophic or keloid scarring occurs when excessive ECM is accumulated, which happens when persistent stimulation of TGF‐β, SMAD3, CTGF, YAP/TAZ, or Wnt signaling occurs. On the other hand, fragile matrix architecture and wound recurrence occur due to insufficient remodeling. Therefore, the determination of whether healing concludes in regeneration, fibrosis, scarring, or chronicity is represented by remodeling, which acts as a key mechanical checkpoint. To achieve functional and scar‐free wound healing, this phase is targeted [25].

## 3 Cellular Mechanism

Wound healing is a well‐synchronized biological reaction that reinstates the structural design of tissue and its function after injury. This process involves various mechanisms like cellular, molecular, mechanical, pathological, genetic, and so forth, that progress through distinctive but intersecting phases: hemostasis, inflammation, proliferation, and regeneration [26]. In the hemostasis phase, after an immediate injury, platelets gather and aggregate in the clot to stop the bleeding. After aggregation, they tend to release numerous growth factors and cytokines to begin the healing process. In addition to this, inflammation occurs when neutrophils and macrophages come together to inhibit the microorganisms present and clear the damaged tissue by releasing more growth factors and cytokines. This regulates the process continuously and leads to the proliferation phase. This is where the barrier cells, like fibroblasts, endothelial cells, and keratinocytes, are activated. The cytokines that are secreted during this phase interact with fibroblast cells, providing structural support and promoting tissue repair. Similarly, they manufacture the ECM components collagen and fibronectin, which form granulation tissue that fills the wound [1]. The endothelial cells recruit immune cells through linking molecules and chemokines by regulating permeability and promoting angiogenesis. So, the keratinocytes divide and migrate to the wound site, where they promote the regeneration of tissues through the MMPs. The disorganized collagen tissues are eliminated, and a new, organized collagen type 1 structure takes over by providing tensile strength to tissues, and the healing ends by clearing out the extra cells. Hence, the healing process is packed and controlled by signaling molecules like growth factors, cytokines, and receptor‐ECM interactions. Likewise, a complete healing process involves the proper management of cellular, molecular, and other mechanisms [27].

### 3.1 Platelets

The protective endothelial layer keeps platelets inactive while they circulate near blood vessel walls under normal physiological conditions. By releasing inhibitory molecules such as prostacyclin and nitric oxide, this healthy endothelium acts as a natural barrier against the formation of clots [28]. Platelet activation occurs when the underlying sub‐endothelial matrix is revealed due to endothelial damage or inflammation. To anchor the platelets and start signaling cascades that result in the formation of an initial platelet plug at the injury site, platelet surface receptors attach to collagen and von Willebrand factor (VWF). The coagulation cascade is then triggered, creating a network of fibrin that keeps the clot stable. In addition to offering a phosphatidylserine‐rich surface for coagulation reactions, platelets also supply prothrombin, polyphosphates, Factor V, Factor XIII, and other essential components to control the process. Simultaneously, soluble mediators such as ADP released from activated platelets can activate platelets via G‐protein‐coupled receptor (GPCR) pathways, which can enhance platelet recruitment and activation through positive feedback loops. In the final stages, vessel integrity is restored by the coordinated release of signaling molecules and interactions between vascular cells, coagulation factors, and platelets [28]. Platelet secretion is crucial for expanding the impact of platelets beyond hemostasis to encompass inflammation, tissue repair, and other processes outside the immediate thrombus environment.

Dynamically moving through the bloodstream, platelets are activated when they come into contact with exposed vascular components such as collagen and VWF at injury sites. Their activation sets off a cascade of signaling events that raise calcium levels in the cytosol, which causes intracellular granules to fuse with the plasma membrane and release molecules that have been stored. Beyond hemostasis, this precisely regulated secretion process plays a crucial role in promoting platelet adhesion and aggregation during clot formation [29]. By drawing in and communicating with immune and tissue cells, platelets' granules release a range of chemokines, cytokines, and growth factors that aid in inflammation, wound healing, and vascular remodeling. As a result, abnormalities in platelet function may lead to excessive clotting (thrombosis), bleeding disorders, or even contribute to the development of inflammatory diseases, immunological dysregulation, and cancer [30].

#### 3.1.1 α‐Granules

Platelet α‐granules, which are the most common secretory organelles (50–80 per platelet), hold a wide range of proteins that are important for hemostasis, inflammation, wound healing, and angiogenesis. They have proteins that stick together, like VWF, fibrinogen, fibronectin, vitronectin, and thrombospondin, which help platelets stick together and form a thrombus. They also contain signaling molecules such as platelet factor 4 (PF4), IL‐8, platelet‐derived growth factor (PDGF), transforming growth factor‐β (TGF‐β), and VEGF. The main chemokines that control the recruitment of inflammatory cells are PF4, β‐thromboglobulin (CXCL7), and RANTES (CCL5) [31]. Multivesicular precursors make α‐granules by taking in materials through biosynthetic (like VWF and PF4) and endocytic (like fibrinogen and IgG) pathways. They are lysosome‐related organelles (LROs) because they have clathrin‐coated membranes and endosomal features. Other examples of LROs are Weibel‐Palade bodies (WPBs), dense granules, melanosomes, and lamellar bodies. Recent evidence contests the idea that α‐granules are uniform; specific subpopulations possess either pro‐ or anti‐angiogenic factors (e.g., VEGF vs. endostatin) and exhibit diverse morphologies, spherical, multi‐vesicular, and tubular. Although α‐granules exhibit structural and functional similarities to WPBs, such as the arrangement of VWF in short tubular helices, the mechanisms that determine their tubular subtypes are not yet fully understood [32].

#### 3.1.2 Platelet Dense Granules

Platelets have dense granules that hold small molecules like ADP, ATP, serotonin (5‐HT), magnesium, and calcium. When platelets are activated, these molecules are released very quickly, much faster than substances from other granules like α‐granules or lysosomes. When they are released, the small molecules and thromboxane A_2_ work together to speed up platelet activation and aggregation through positive feedback loops. The ADP‐P2Y_12_ receptor pathway is a key part of this process, and it is necessary for normal blood clotting. Mice that don't have this receptor have big problems with platelet adhesion, integrin activation, and secretion. Another study on mice that lacked the Munc13‐4 protein, which is necessary for the release of dense granules, found that their platelets couldn't properly secrete ADP [33]. This made it hard for the platelets to stick together and form clots. Adding ADP from the outside, on the other hand, partially restored their platelet function, confirming that the ADP released from platelets (not circulating ADP) is what makes clots form properly. Interestingly, these mice were shielded from stroke‐related clot progression without experiencing heightened cerebral hemorrhage, indicating that ADP and P2Y12 signaling may have divergent functions in normal hemostasis versus pathological thrombosis. In general, ADP's role in platelet activity seems to be more complicated than previously thought. It affects not only clot formation but also larger procoagulant and thrombin‐activating pathways [34].

### 3.2 Neutrophils

Neutrophils are the first cellular response to occur immediately after an injury. They act as a barrier and as a defense mechanism in the early period of wound healing. These are the fastest responders in the healing process, which promotes angiogenesis via complete coordination of endothelial cells. Neutrophils reach the injury site through the attraction of chemoattractants, which include tumor necrosis factor alpha (TNF‐α), IL‐1, and lipopolysaccharide (LPS). After reaching the wound site, these neutrophils release immediate antimicrobial activity, which consumes microorganisms and cellular debris via phagocytosis by launching ROS activity, peptides with antimicrobial properties, and proteolytic enzymes. These, in turn, safeguard the wound's surroundings, thus promoting rapid healing. Likewise, it leads to collateral damage if the infection persists for a prolonged period. NETs, a web‐like structure composed of DNA and protein peptides released by neutrophils, control and inhibit the growth of microorganisms and assist other immune cells in clearing them out. But in the case of diabetes, excessive NETs affect and slow the healing of wounds. Alongside, the inflammation in diabetes is also complex, where abnormal inflammation results in delayed healing by making the wound more complicated. In the early period, neutrophils connect with other cells and increase their attachment through packed behavior [35].

#### 3.2.1 Properties of Neutrophils

In simple terms, neutrophils have numerous roles in wound healing, as they act as investigator, dividers, antimicrobial agents, signaling controllers, communicators, modifiers, and removers. Neutrophils act as a signaling hub that enhances the wound environment through inflammatory properties. It connects with fibroblast cells, platelets, and endothelial cells, leading to angiogenesis, coagulation, and rapid ECM remodulation, through which it assesses the future phases of healing [16]. A recruited neutrophil immediately releases leukotriene B4 (LTB4), a lipid mediator, and myeloperoxidase and elastase, signaling proteins, when it encounters microorganisms. This attracts more neutrophil cells and enhances the inflammatory responses, but it needs to be downregulated to improve the repair mechanism of wounds [36]. So, when the unwanted materials are cleared, the neutrophil undergoes changes like apoptosis, which reduces its lifetime. The neutrophils, which are dead, are engulfed by the macrophages through a process called efferocytosis, simultaneously consuming the dead cells as well as releasing anti‐inflammatory signals that attract more macrophages and alter the process into a repetitive system. Sometimes this process can be reversed, where it enters the bloodstream, which resists the gathering of inflammatory cells and modulates tissue integrity. Similarly, when these immune cells stay in the wound site or the biological system for a prolonged period, they generate dangerous materials like NETs, ROS, and proteases. This results in breaking down the nearby factors of the wound site, disrupting healing processes like fibroblast function, new blood vessel formation, and skin cell movement. As a result, wounds fail to heal and become chronic [37].

### 3.3 Macrophages in Wound Healing

The essential tissue function of M1 macrophages was restored by monocytes with higher production of inflammatory cytokines like IL‐1β, IL‐6, and TNF‐α. When an injury occurs, the tissues often strive for support from two different types of macrophages: the old ones that were developed since birth, and others that were recruited from the blood [38]. Macrophages are essential cells that guide how the wound site should heal, because they are present throughout the biological system and act according to every condition. Some researchers suggested that macrophages work according to the different functions of the diverse organs [39]. Likewise, they also yield ROS and MMPs. Macrophages arise from two different sources, namely Langerhans cells and dermal macrophages. The damage‐associated molecular patterns (DAMPs), released by the cells due to delayed healing, and pathogen‐associated molecular patterns (PAMPs) situated on the pathogens, were recognized by the macrophages. These were identified by pattern recognition receptors (PRRs) like Toll‐like receptors (TLRs) [40]. Complete resolution of inflammation is essential, and it happens when the pathogens, debris, dead cells, and dead neutrophils are removed through phagocytosis, primarily by M1 macrophages. When the dead neutrophils are consumed through phagocytosis, it initiates the macrophage to transition from M1 to M2 form. However, this M2 type promotes fibroblast growth, vascular growth, tissue regeneration, and ECM rearrangement [41]. So, when inflammation occurs, these macrophages move into the wound and take on either M1 or M2 forms with the help of signals like growth factors, cytokines, lipids, and oxidants, which are released by them [42]. VEGF, PDGF, FGF, and TGF‐β are the mediators that M2 macrophages secrete.

When it reaches the remodeling phase, macrophages continue to improve the repair process by regulating the balance between collagen deposition and degradation, which strengthens the ECM matrix. So when this process is completed, the macrophages sometimes undergo apoptosis or are returned to the bloodstream, which ends the inflammatory stage and promotes a rapid regenerative process. The function of abnormal macrophages disrupts the normal process of wound healing. This affects the macrophage transition from M1 to M2 form, leading to prolonged inflammation and delayed healing. Similarly, when the prolonged M1 form persists, it increases the chance of damaging the newly synthesized fibroblast cells [17].

### 3.4 Keratinocytes in Wound Healing

Following a wound, keratinocytes have an important role in tissue regeneration. When a wound occurs, the skin damage exposes the internal layer to the external environment, where pathogens have easy access to the internal layer, disrupting the underlying tissues, ultimately leading to dryness and dehydration. In order to strictly inhibit the entry of pathogens, the keratinocytes restore normal function by stimulating the biochemical signaling released by platelets and immune cells [43]. The biochemical signals, like epidermal growth factors, transforming growth factors, and keratinocyte growth factors, initiate the signaling cascades through other pathways like EGFR/MAPK and PI3K/Akt, resulting in the proper arrangement of the ECM with increased cell numbers [44]. The keratinocytes present on the wound site undergo morphological and functional transitions where they activate from an inactive state and move by continuously dividing. This enhances ECM remodeling, which creates a pathway for cell movement. This occurs due to the multiplication of keratinocytes, which cross the fibrin and fibronectin‐rich layer with the help of integrins and MMPs. When the keratinocytes' secretion process continues, they stimulate the production of fibroblasts and endothelial cells, which link and rebuild the epidermal and dermal layers. These secretions are due to stimulation of cytokines like IL‐1, TNF‐α, and VEGF, which occur in the proliferative phase [45].

However, when the keratinocytes divide and reach the center of the wound, multiple epidermal tissues will be synthesized, where the new cells move upward from the basal layer, helping re‐form the outer skin by enhancing the barrier function. Later, in order to control collagen deposition and to prevent the wound from scarring, the keratinocytes offer a signal to fibroblast cells through paracrine signaling [46]. Any changes in keratinocyte division often result in delayed healing, which ultimately leads to the formation of a chronic ulcer and abnormal scarring. Hence, keratinocytes serve as an active ingredient in controlling inflammation and tissue regeneration during the repair mechanism [47].

### 3.5 Fibroblasts in Wound Healing

**Figure 2:**
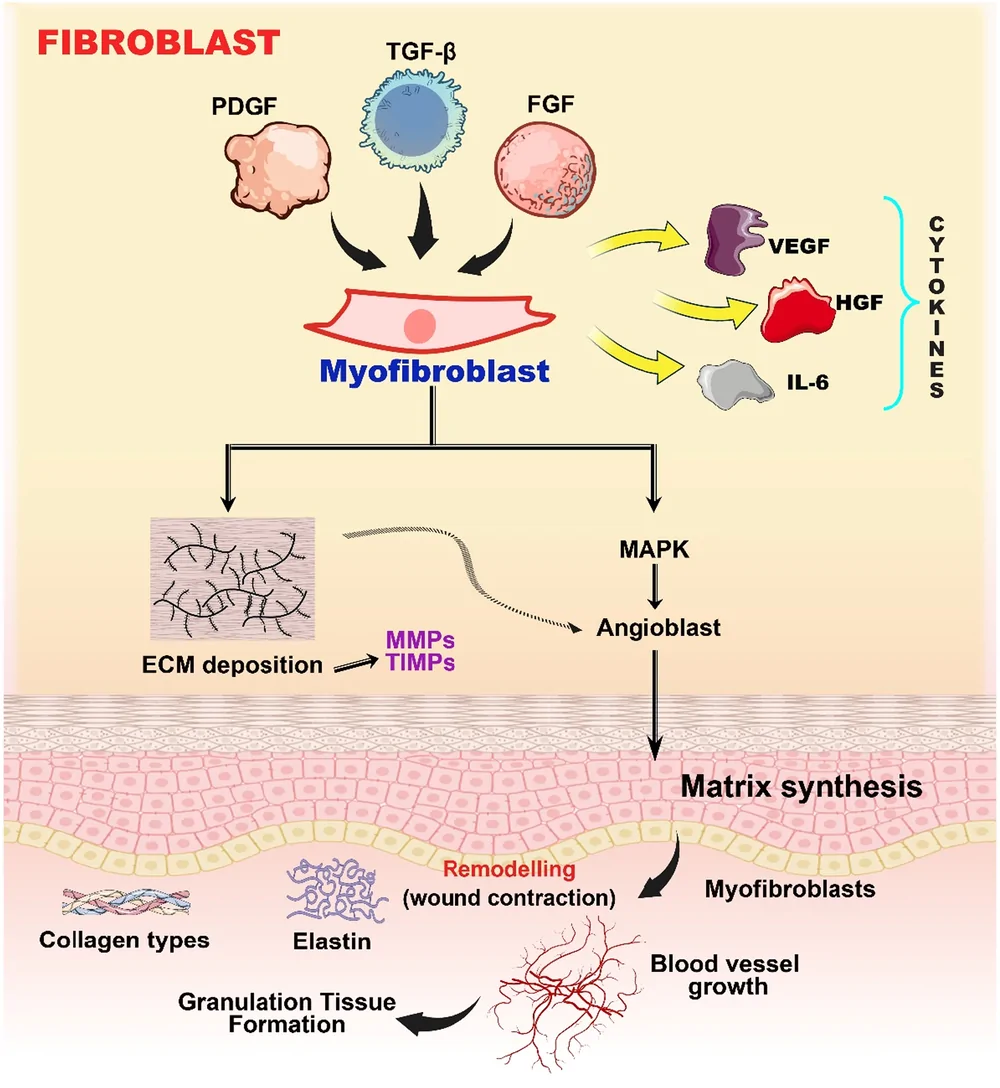
Describes the fibroblast to myofibroblast conversion and its complex role in tissue regeneration.

During wound healing, the fibroblast cells, otherwise called mesenchymal cells, play a crucial role in synthesizing granulation tissue and ECM remodeling. They begin their function following signals stimulated by PDGF, TGF‐β, and FGF growth factors, which are produced by platelets and macrophages. This usually happens around 48–72 h after injury. After receiving the signals, the fibroblast activates, then multiplies and travels to the wound site, where it starts to produce ECM components that comprise type 1 collagen and type 3 collagen, elastin, fibronectin, and proteoglycans. Therefore, the synthesized matrix acts as a framework for angiogenesis and regeneration with improved mechanical stability [48]. Figure 2 explains the multifaceted interactions performed to enhance the regeneration of tissue. Alongside, it also produces MMPs and tissue inhibitors of metalloproteinases, which balance ECM synthesis as well as degradation, ensuring complete tissue remodeling. The fibroblasts expand and mature into myofibroblasts through TGF‐β/Smad and MAPK signaling pathways, aiding wound contraction. However, fibroblasts promote cell signaling through the secretion of cytokines like VEGF, HGF, and IL‐6, which rapidly promote angiogenesis and division of epithelial cells. Sustained fibroblast production is crucial during the remodeling phase, because this is the phase where the collagen is reorganized and restores its tensile strength. Hence, abnormal fibroblast activity, like increased TGF‐β/Smad production, ultimately results in fibrosis, abnormal scarring, even keloid or hypertrophic scars [49].

## 4 Molecular Mechanisms

Delayed healing or abnormal wounds are the main drawback that leads to chronic wounds. Chronic wounds take a prolonged period to completely heal. However, it causes a sudden change in the normal wound healing process by initiating frequent infections, which affects inflammation, growth of nerve cells, vascular tissues, and necrotic tissues. These factors are highly seen in the individual suffering from diabetes, rheumatoid arthritis, pressure ulcers, malnutrition, venous ulcers, or bedridden individuals. Though the chronic wound produces high levels of proteolytic activity, almost 200‐fold higher than the acute wound, when this activity fails in proteolytic degradation, the cells completely stop stimulating the healing processes. Hence, identifying suitable protease inhibitors is required for their application in combination with exogenous mitogen therapy [50].

### 4.1 Growth Factors and Cytokines

The essential biochemical intermediary that controls the cellular and molecular behavior of wound healing is growth factors and cytokines. The essential biochemical intermediary that controls the cellular and molecular behavior of wound healing are growth factors and cytokines. Table 2 comprises the various growth factors and their complex roles in the molecular mechanism. As the process of healing wounds is complex, it is maintained by the functioning of signaling networks, which ultimately initiate the formation and migration of various growth factors, chemokines, and cytokines. However, some specific growth factors have a major role in promoting wound healing, such as IGF, VEGF, PDGF, TGF‐β, connective tissue growth factor (CTGF), FGF, granulocyte macrophage colony‐stimulating factor (GM‐CSF), tumor necrosis factor‐α, and the interleukin family. These factors stimulate the activation of various cell types, including fibroblasts, epithelial cells, keratinocytes, and vascular endothelial cells, which promote advanced healing processes, including granulation and subsequent epithelialization [61].

**Table 2:** Critical role of growth factors in the molecular mechanism of wound healing.

S No	Growth Factors	Cells involved	Key role in wound healing	Major pathway influencing the outcome	Reference
1	Vascular Endothelial Growth Factor (VEGF)	Keratinocytes, fibroblasts, and macrophages	Increases the permeability of the bloodstream, thus promoting angiogenesis.	VEGFR‐driven PLCγ/PKC‐mediated signaling for endothelial division	[51]
2	Platelet‐Derived Growth Factor (PDGF)	Platelets and macrophages	Attracts fibroblast cells and smooth muscle cells to the wound site, which promotes the formation of tissue granules	Triggers PI3K/Akt and MAPK signaling to improve cell movement and division	[52]
3	Fibroblast Growth Factor (FGF)	Fibroblasts, endothelial cells, and macrophages	Increases the production of fibroblasts, which promote the angiogenesis process and collagen formation.	FGFR‐activated ERK pathway stimulates fibroblast proliferation and ECM formation.	[53]
4	Transforming Growth Factor‐Beta (TGF‐β)	Platelets, keratinocytes, and macrophages	Highly controls the inflammation process, which encourages fibroblast division and collagen formation.	Dual regulation of ECM deposition via Smad‐dependent and Smad‐independent mechanisms	[54]
5	Keratinocyte Growth Factor (KGF)	Keratinocytes and fibroblasts	Promotes keratinocyte formation, which highly initiates wound closure	Activates STAT and MAPK signals by binding to FGFR2 receptors.	[55]
6	Nerve Growth Factor (NGF)	Macrophages and keratinocytes	Increases the formation of nerve cells, which ultimately increases vascular growth.	TrkA signaling pathways promote angiogenic and neurogenic repair.	[56]
7	Epidermal Growth Factor (EGF)	Keratinocytes, platelets, and keratinocytes	Promotes the movement of epithelial cells, which increases the regeneration of tissue.	EGFR activation triggers MAPK and ERK cascades for cell proliferation	[57]
8	Insulin‐like Growth Factor (IGF)	Keratinocytes, fibroblasts, and macrophages	Improves the repair mechanism by promoting cell division and protein formation.	Activates the PI3K/Akt pathway to promote survival and matrix deposition	[58]
9	Connective Tissue Growth Factor (CTGF)	Endothelial cells and fibroblasts	Facilitates matrix remodeling and myofibroblast division.	TGF‐β‐induced Smad pathway mediates key events in tissue development and remodeling.	[59]
10	Hepatocyte Growth Factor (HGF)	Fibroblasts and mesenchymal cells	Stimulates epithelial and endothelial cell death, which enhances wound shrinkage.	c‐Met signaling drives mitogenic and survival pathways via MAPK and PI3K/Akt pathways	[60]

#### 4.1.1 Insulin‐Like Growth Factors (IGFs)

IGFs are growth factor that has a strong structure that resembles insulin, especially proinsulin. The liver is the specific site for the formation of this growth factor; however, the IGFs formed in the liver act in an endocrine manner, while IGFs from numerous sites in the body act in an autocrine or paracrine manner [62]. This displays a wide range of gross physiological effects, and it plays a major stimulatory role in the cell cycle and contributes to sustained and uncontrolled cell growth with cancer cell characteristics. Hence, every mammalian cell type exhibits this surface IGF receptor. When the essential level of IGFs is not present, it leads to osteoporosis in individuals diagnosed with bone marrow complications. Moreover, IGF‐1 mediates most growth‐promoting effects, such as treating dwarfism and stimulating renal function. IGF‐2 is specifically produced by tissue at vascular interfaces with the brain, and both IGF‐1 and IGF‐2, along with insulin, play a crucial role in the nervous system. They initiate the growth and development of various neuronal residents and promote neurotrophic effects, which are utilized in the treatment of various neurodegenerative diseases.

#### 4.1.2 Epidermal Growth Factor (EGF)

EGF is a 6‐kilodalton, 53‐amino acid, unglycosylated peptide and the first growth factor discovered [63]. It has been shown to exhibit a prevailing mitogenic effect on many cell types, particularly on endothelial cells, epithelial cells, and fibroblasts. Hence, the skin and the wounds on the skin layer appear to be EGF's major physiological target. During the wound healing process, EGF plays a vital role in promoting re‐epithelialization, cell proliferation, and restructuring of tissues. Keratinocytes, fibroblasts, and endothelial cells express a special transmembrane tyrosine kinase receptor, the epidermal growth factor receptor, where EGF exerts its biological function by binding to it. Binding of EGF on their targeted EGFP results in series of process such as receptor dimerization, auto‐phosphorylation of intracellular tyrosine residues, following by activation of downstream pathways such as PI3K/Akt, MAPK/ERK, JAK/STAT and PLCγ [64]. Τhese pathways collectively contribute in promoting functions such as keratinocyte survival, collagen synthesis, cytoskeletal reorganization, DNA replication and cellular migration which is essential for epithelial gap closure. In the proliferative phase of wound healing, EGF plays a vital role in restoring epithelial continuity by inducing keratinocyte migration across the wound bed [65]. In addition to strengthening epithelial adhesion and tissue integrity, it stimulates collagen IV expression, laminin expression, and integrin expression in the basement membrane. Aside from EGF, TGF‐β, IL‐10, and PDGF, signaling also affects fibroblast behavior indirectly, ensuring coordinated ECM and immune system formation. Moreover, EGF protects mitochondria and increases cellular stress resistance in diabetic and ischemic wounds, where oxidative stress impairs keratinocyte function [66]. Biofilms, protease imbalances, or hyperglycemia are frequently responsible for downregulating EGFR expression in chronic wounds, which delays epithelialization. A study conducted by Li and his colleagues found that calcium silicate stimulates epidermal stem cell proliferation and migration by activating the EGF/EGFR/ERK signaling axis [67]. Experimental wounds from mice with excisional wounds showed significantly faster closure as well as thicker neo‐epidermal tissue than controls (*p* < 0.001). An EGF/EGFR activation strategy induced by biomaterials is identified as an efficient mechanism for improving epithelial regeneration in chronic wounds by this study.

#### 4.1.3 Platelet‐Derived Growth Factor (PDGF)

PDGF is identified as the major growth factor that is synthesized by platelets, and it plays an important role in healing wounds. The growth factor is released onto the wound site by activated platelets, which act as chemoattractants or mitogens for various cells, controlling and initiating tissue repair [68]. Likewise, it displays a signaling effect on fibroblasts, glial cells, and smooth muscle cells. Active PDGF is a dimer, with two constituent polypeptides, A and B, and three active PDGF isoforms: AA, AB, and BB. According to some in vitro studies, PDGF BB and AB dimeric isoforms are the most potent forms in wound healing. It has been proven that PDGF is an essential growth factor in wound healing, and both human trials with this BB isoform showed higher healing rates in chronic wounds by stimulating remarkable healing acceleration [69].

#### 4.1.4 Vascular Endothelial Growth Factor (VEGF)

VEGF is a subfamily of the PDGF family, which in turn is from a 16‐knot growth factor, and is a homodimeric glycoprotein with two receptor‐binding domains (RBD) and two heparin‐binding domains (HBD) [70]. The RBD binds to its respective receptors, whereas the HBD facilitates co‐receptor interaction, such as NRP‐1 in the case of VEGF‐A protein, which is involved in promoting angiogenesis and vasculogenesis. Likewise, these proteins are involved either directly or indirectly in bone formation, hematopoiesis, and wound healing. The VEGF growth factor is further classified into five different classes, such as VEGF‐A, VEGF‐B, VEGF‐C, VEGF‐D, and PLGF. The corresponding genes for VEGF‐A were found on chromosome 6 at 6p21.3 locations [71]. Likewise, the receptors corresponding to VEGF‐A are VEGFR‐1 (FLT‐1) and VEGFR‐2 (FLK‐1/KDR), where VEGFR‐2 mediates all functions. VEGF‐A upregulates the production of nitric acid and stimulates endothelial cell division, mitosis, MMPs, and αVβ3 integrin activity. On occasion, VEGF‐A is related to diseases like age‐related macular degeneration (AMD), where the retina secretes extra VEGF‐A, which ultimately causes growth and leakage of VEGF into blood vessels [72].

#### 4.1.5 Fibroblast Growth Factor (FGF)

FGF is a family of 20 proteins, which are numbered consecutively from FGF‐1 to FGF‐20. They exhibit a molecular mass in the region of 18–28 kDa and induce a series of mitogenic, angiogenic, and chemotactic responses. Altogether, they have 140 amino acids, with the central core highly homologous among all family members. All forms of FGFs tightly bind heparin and heparin‐like glycosaminoglycans found in the ECM matrix [73]. They work as master regulators, controlling the crucial wound healing process via particular receptor‐mediated signaling pathways, which ultimately restore tissue quality.

FGF2, in its basic form, plays a crucial role in activating fibroblasts, keratinocytes, and endothelial cells, which further connects with FGF receptors like FGFR1–FGFR4, producing intrinsic tyrosine activity [74]. The essential processes, angiogenesis, collagen formation, and migration of fibroblasts, were activated by FGFR dimerization, which promotes crucial signaling pathways like RAS/MAPK, PI3K/Akt, and STAT, by binding with the ligand. Similarly, FGF enhances fibroblast migration, collagen production, and promotes blood vessel growth, which is essential for oxygen and nutrient delivery to the wound site. It also enhances the movement of keratinocytes and epithelial gap closure, rapidly promoting tissue generation from the inflammation phase to the proliferative phase. Studies on diabetic and ischemic ulcers often have low FGF expression, which contributes to impaired angiogenesis and delayed fibroblast activation, resulting in chronic non‐healing wounds [75].

FGFs also function as potent angiogenic mediators, provoking vascular maturation, endothelial sprouting, and capillary tube formation. In the hypoxic environment, FGF‐driven angiogenesis helps in the continuous supply of nutrients and oxygen diffusion, alongside promoting fibroblast function and granulation tissue formation. Besides regulating ECM turnover, FGFs regulate MMP/TIMP balance, which facilitates controlled remodeling and prevents excessive fibrosis [76].

Keratinocyte migration and re‐epithelialization are supported by FGFs through FGFR2‐mediated signaling. They also promote basement membrane reconstruction by enhancing laminin and fibronectin deposition [77]. FGFs also facilitate epigenetic modulation, guiding chromatin remodeling and gene transcription for repair‐associated cells. Biomaterials with controlled release properties and hydrogel‐based FGF delivery systems have shown promising results in preclinical and clinical wound models. However, FGFs also suffer from certain limitations in attaining their therapeutic efficacy due to their short half‐life and instability in protease‐rich environments. Advancements in FGF delivery systems, such as nanocarrier‐based FGFs, gene‐modified FGF vectors, and ECM‐mimicking scaffolds, are considered major alternatives to overcome these limitations and to achieve enhanced bioavailability and targeted delivery [78]. Thus, FGFs are considered to be a major central regulator of wound healing, with strong potential for precision therapeutics and regenerative medicine.

#### 4.1.6 Transforming Growth Factors (TGFs)

TGFs are another family of polypeptide signals that includes TGF‐α and TGF‐β. These are initially synthesized as integral membrane proteins, and the proteolytic cleavage releases the soluble growth factor, which displays a high degree of amino acid identity with EGF. Hence, it exerts its biological effects by binding to the EGF receptor. TGFs are produced by numerous tumor cell types, for which they act as an autocrine growth factor. These are further classified into three different growth factors, like TGF‐β1, TGF‐2, and TGF‐3, which are the products of distinct genes, but they all display adjacent identity [79]. However, most body cells produce TGF‐β, either separately or in combination, where it tends to induce transformation of fibroblast cell lines in the beginning, and later, they are known to be a pleiotropic cytokine that is proficient at inhibiting the cell cycle and cell growth of several cell types, like epithelial and hematopoietic cells which induce the formation of ECM proteins and modulate the expression of matrix proteases. It acts as a powerful chemoattractant for monocytes and fibroblasts, which have a major role in tissue remodeling, wound repair, and hemopoiesis [80].

#### 4.1.7 Neurotrophic Growth Factors (NGFs)

NGFs constitute a group of cytokines that regulate the development, maintenance, and survival of neurons, both in the central and peripheral nervous systems. An important subfamily of neurotrophic factors is the neurotrophins [81]. Contrary to traditional views, many neurotrophic factors are synthesized not only by neuronal target cells but also by non‐neuronal target cells and influence cells other than neurons, like NGF produced by mast cells, and influence numerous cells of the immune system. Moreover, comprising various growth factors, numerous cytokines are now known to influence neuronal cells. A lot of NGFs endure definite neuronal residents whose death underlies various neurodegenerative diseases. This increases the possibility of providing the essential molecules for effective treatments for neurodegenerative diseases in the future [82].

#### 4.1.8 Importance of Preserving the Mitochondrial Process for Regulating the Growth Factor‐Mediated Wound Healing

Mitochondria play a significant role in promoting the biological processes of growth factors in wound healing. Though they have an important function in regulating ATP production, they also play a significant role in maintaining oxidative balance and producing energy for the migration of keratinocytes, fibroblast division, endothelial cell function, and ECM deposition. Hence, these processes are very important for regulating the progression of the above growth factors [83]. Frequently, mitochondrial reactive oxygen species (mtROS) act as an intracellular signaling molecule and accelerate growth factor‐based cellular reactions, and the advancement of controlled mtROS enhances vascular generation, cell division, and new tissue formation. However, mitochondrial dysfunction occasionally promotes higher ROS generation, which ultimately increases oxidative stress and affects the production of cellular proteins, lipids, and DNA. Alternatively, the increased oxidative stress alters the normal process of growth factors by affecting and minimizing the production and promotion of angiogenesis, fibroblast cells, and ECM deposition. So reducing the inflammatory responses and retaining the homeostasis of mitochondria is crucial for maintaining the biological activities of growth factors throughout the healing process [84]. Researchers stated that mitochondria regulate the pathways under physiological conditions that decrease intracellular redox signaling, thus retaining the essential metabolism required for cellular processes and healing of tissues. Mitochondrial DNA is sometimes released into the cytoplasm due to infection‐, chronic inflammation‐, hyperglycemia‐, and ischemia‐induced alterations in mitochondrial ROS production. The impairment not only causes ROS production but also activates several inflammatory signaling pathways, especially the NF‐ κB signaling pathway and the NLRP3 inflammasome. Excessive oxidative stress damages the mitochondrial membrane, leading to unstable membrane function, reduced ATP synthesis, and altered migration and division of keratinocytes and fibroblasts, thereby reducing tissue regeneration and ECM formation [85].

## 5 Signaling Pathways Involved in Wound Healing

### 5.1 The AKT/PKB Signaling Pathway in Wound Healing

**Figure 3:**
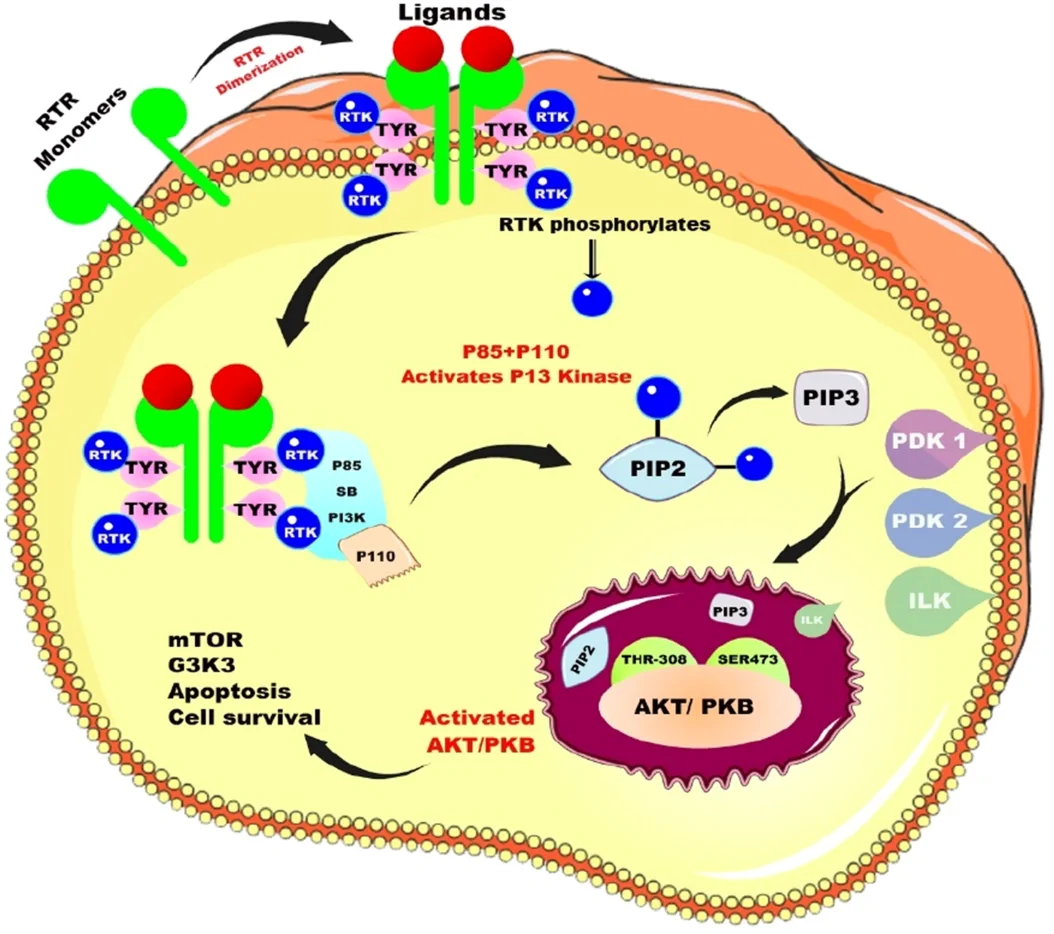
Overview of receptor tyrosine kinase‐mediated activation of the PI3K–AKT signaling pathway.

The AKT/PKB signaling pathway is also called the PI3–AKT signaling pathway, which promotes cell survival, growth, division, movement, angiogenesis, and metabolism [86]. The most important proteins or molecules involved are AKT, or PKB, which is nothing but protein kinase B, and P13K or P13, which is phosphoinositide‐3‐kinase. However, the essential cell membrane receptor involved is RTK, also called receptor tyrosine kinase. So, in the cell membrane, the receptor is present in the form of an RTK monomer, and before signaling, it would be in an unphosphorylated state at the tyrosine residues. When the ligands enter and bind to these monomers, it initiates the dimerization of the monomers, where ligands get close enough to bind with each other. Therefore, the dimerization stimulates the kinase activity of the RTK, with which the RTK cross‐phosphorylates its own residues. However, different proteins may bind to a phosphorylated domain, like insulin receptor substrate 1 (IRS 1), which binds to the activated IGF1 receptor for simplification. Then this receptor was replicated as a dimer receptor, which bound IRS‐1 and served as a binding and activation site for the PI3 kinase [87]. In addition, this PI3 kinase binds directly to a phosphorylated receptor tyrosine kinase. PI3 kinase activation begins with the small membrane‐bound Ras‐GDP, which is converted to active GTP‐bound Ras. Active Ras then directly binds to and activates PI3K. Alternatively, PI3K can be activated by binding to the GTP‐bound form of the membrane‐bound protein Ras. Active P13K catalyzes the formation of membrane‐bound phosphatidylinositol (3,4,5) triphosphate (PIP3). The second messenger PIP3 activates the serine‐threonine kinase [88]. Active AKT phosphorylates many proteins, such as 3‐phosphoinositide‐dependent kinase 1 (PDK1), and activates mammalian target of Rapamycin C2 (mTORC2), which leads to the inhibition of apoptosis and to the activation of translation and proliferation [89]. Figure 3 explains the mechanistic pathway of PIP3/Akt signaling following RTK stimulation.

### 5.2 The Wnt/β‐Catenin Pathway in Wound Healing

**Figure 4:**
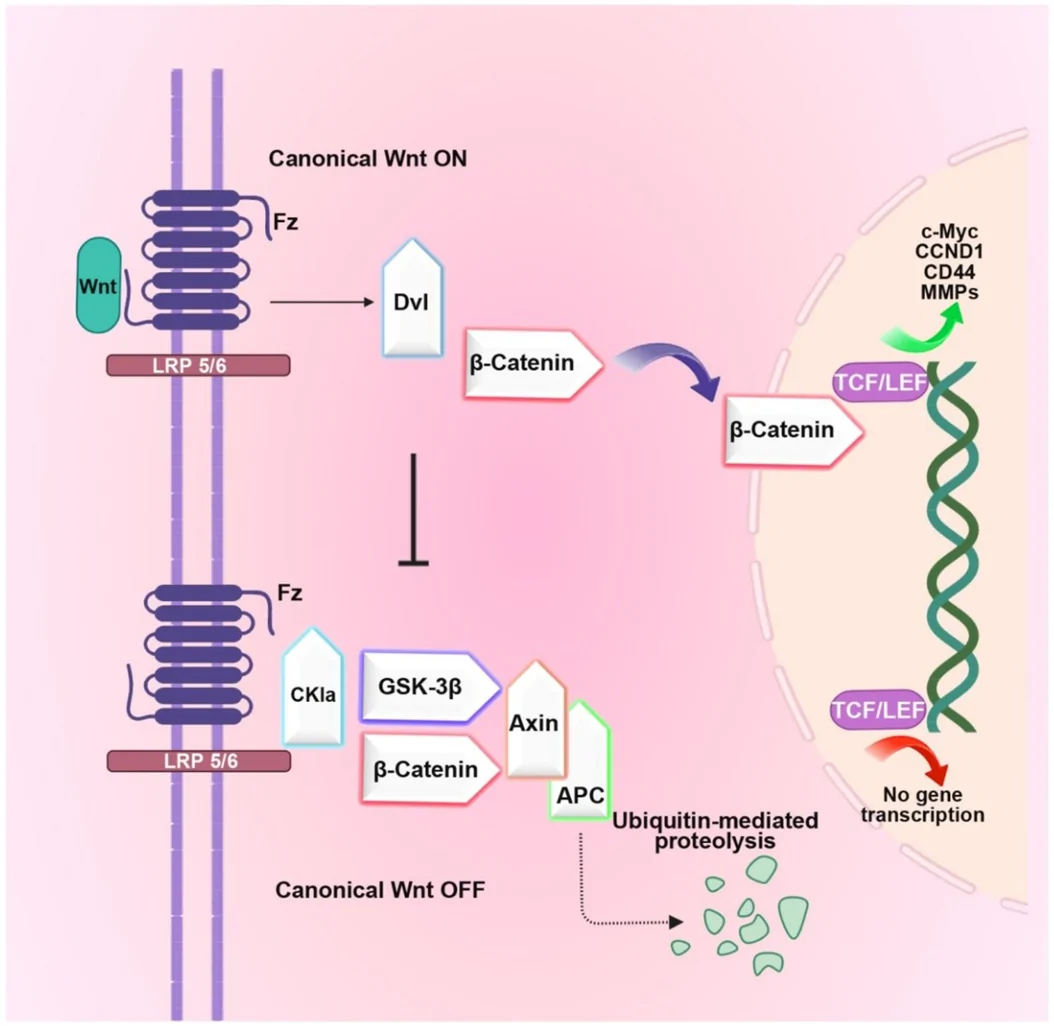
Wnt/β‐catenin pathway in wound healing: Wnt‐ON and Wnt‐OFF circumstances.

This pathway is essential for the developmental process of tissues and consists of two transmembrane receptors, such as frizzled G protein and lipoprotein receptor‐related protein (LRP). These proteins receive ECM signals simultaneously in the form of Wnt proteins. The Wnt pathway is also called the β‐catenin pathway because the β‐catenin molecule, located in the cytosol, transduces the signal and regulates the Wnt signal in the pathway. In the absence of a Wnt signal, the Wnt receptors are positioned in the cytoplasm without a signal [90]. Then β‐catenin is bound by a group of proteins, including APC, axin, GSK3, and CK1. These proteins phosphorylate β‐catenin, which makes it unstable and marks it for destruction by the proteasome. Therefore, the entry of β‐catenin into the nucleus is prohibited due to its instability and low protein levels. In the nucleus, there are transcription factors called LEF1/TCF that are present, which bind to the DNA and control the expression of Wnt‐responsive genes. However, without β‐catenin, these factors are inactive, as they are associated with a co‐repressor protein called Groucho, which prevents gene transcription [91]. Figure 4 illustrates the canonical Wnt/β‐catenin signaling pathway, expressing the molecular differences between Wnt‐OFF and Wnt‐ON conditions.

Furthermore, in the presence of the Wnt signal, the receptors Frizzled and LRP bind Wnt and activate it, which leads to the stabilization of β‐catenin. Firstly, the cytoplasmic tail of the LRP receptor is phosphorylated by GSK3 and CK1, which creates a binding site for axin. Axin is a scaffold protein that typically holds the β‐catenin degradation complex together. When axin binds to LRP, it is removed from the complex, causing the complex to fall apart, indicating non‐degraded or phosphorylated β‐catenin, which then accumulates in the cytoplasm. Then, β‐catenin translocates to the nucleus, where LEF1/TCF and Groucho present as a complex and act as a repressor of transcription. Later, β‐catenin binds to LEF1/TCF and displaces Groucho, which converts LEF1. TCF from a repressor agent to an activator agent of gene transcription. The genes that are activated by β‐catenin and LEF1/TCF are involved in various wound healing processes, like cell differentiation, proliferation, and migration [92].

### 5.3 Mitochondrial Dysfunction in Chronic Wounds and Inflammatory Signaling Pathways

Mitochondrial dysfunction plays a major role in altering signaling pathways through excessive oxidative stress, which increases ROS generation and thus modifies the production of lipids, cellular proteins, and DNA. It is essential to retain mitochondrial homeostasis in wound healing [93]. Though temporary mitochondrial dysfunction occurs right after the injury, it is normal, whereas stubborn and prolonged mitochondrial dysfunction is critical and often acts as a hallmark of chronic wounds and scarring. This dysfunction limits the progression of the inflammatory process to the proliferative phase and delays the remodeling phases of wound healing by working as a vicious cycle. These cycles are due to chronic inflammatory conditions, such as diabetic ulcers, chronic ulcers, and ischemic wounds, followed by persistent oxidative stress, altered mitochondrial metabolism, a prolonged inflammatory phase, and reduced angiogenic activity. The foremost limitation of mitochondrial dysfunction is the extreme accumulation of defective mitochondria that increases the production of oxidative stress while altering cellular functions. The accumulated mitochondria gather in keratinocytes, immune cells, fibroblasts, and endothelial cells due to prolonged infection, inflammation, metabolic stress, and delayed biological activity [93].

Similarly, it releases the mitochondrial damage‐associated molecular patterns (mtDAMPs), which include mitochondrial DNA (mtDNA), ATP, cardiolipin, *N*‐formyl peptides, and mitochondrial ROS. Likewise, the NLRP3 inflammasome has gained significant attention due to its significant role in wound pathogenesis compared to various inflammatory pathways activated by mitochondrial disruption [94]. Although it delays wound closure and reduces the production of wound‐healing mediators such as VEGF, EGF, and MMP‐9, NLRP3 has been identified as an essential regulator of skin wound healing. It also controls contact between macrophages and myofibroblasts, highlighting its role in coordinating inflammatory responses with ECM remodeling and tissue repair [95]. Increased ROS and the release of mtDNA in the cytoplasm promote the accumulation of the pro‐inflammatory cytokines IL‐1β and IL‐18 [94]. Macrophages from diabetic human and mouse wounds have been shown to have prolonged NLRP3 inflammasome activity, which contributes to the chronic inflammatory response and poor healing features of diabetic wounds. Significantly, reducing inflammasome activity enhanced wound healing, promoted a change in macrophage phenotypes from pro‐inflammatory to repair‐associated, and increased the expression of pro‐healing growth factors [96]. Recent studies indicate that mitochondrial damage can worsen wound healing by increasing ER‐mitochondrial interactions, which stimulate NLRP3 inflammasome activation and increase IL‐1β production. In contrast, reducing ER‐mitochondrial contact suppressed NLRP3 activation and restored wound closure, highlighting the critical role of ER‐mitochondrial communication in controlling inflammation during wound healing [97]. Similarly, prolonged and stubborn inflammasome activation is often observed in diabetic wounds, where the hyperglycemic index and oxidative stress are noted to be increased and stimulate inflammasome signaling. A study stated that alteration in the communication between mitochondria and the endoplasmic reticulum promotes NLRP3 inflammasome signaling. Furthermore, the process of new blood vessel formation was also significantly affected by mitochondrial dysfunction, acting as a critical factor for chronic wound impairment [94]. During the proliferative phase of healing, the endothelial cells require a large amount of blood supply, which then helps in the migration and division of cells and tissues to the remodeling phase. However, dysfunction alters the usual process by minimizing ATP synthesis and oxidative phosphorylation, while generating excessive ROS. This delays the transition from the proliferative phase to the remodeling phase, affects the migration and proliferation of endothelial cells, decreases VEGF‐mediated angiogenic signaling, and promotes blood vessel formation inside the wound bud itself [98].

Hence, these observations indicate that mitochondrial dysfunction acts as a central molecular agent controlling chronic inflammation, oxidative stress, damaged angiogenesis, fibroblast disability, and altered ECM. This concludes whether the mitochondrial dysfunction coordinates the numerous interrelated pathways that regulate healing or is trapped in the inflammatory phase itself [99].

### 5.4 Effect of Pathogen‐Induced Mitochondrial Dysfunction in Wound Healing

In chronic wounds, constant bacterial infection is the other major concern for delayed healing, where pathogens like *Staphylococcus aureus* and *Pseudomonas aeruginosa* colonize the wound area. These pathogens not only affect inflammation but also interact with the mitochondrial membrane, thus affecting the cellular mechanism of tissue regeneration and healing. The pathogens release toxic factors, which initiate the production of mtROS that disrupts the membrane of mitochondria, alters the synthesis of ATP, and promotes mitochondrial DNA damage [100]. This affects the synthesis of keratinocytes, angiogenesis, proliferation, migration of immune cells, and ECM deposition. The activation of signaling pathways like NF‐kB and NLRP3 inflammasome modifies the inflammatory responses and inflammatory cytokines such as IL‐1β, IL‐6, and TNF‐α [100].

The toxic factor called pyocyanin, produced by *P. aeruginosa*, damages the respiratory pathway of mitochondria and increases the level of oxidative stress [101]. Likewise, the pathogen *S. aureus* produces toxins that disrupt the mitochondrial cell wall, promoting host cell apoptosis. These ultimately increase and affect the essential properties that promote wound healing and tissue regeneration [102].

## 6 Genetic Mechanism

Like other mechanisms, the genetic mechanism plays a pivotal role in wound healing. It can control and express the essential genes responsible for every phase of wound healing. This mechanism also expresses the genes that are important in promoting inflammation, tissue repair, angiogenesis, and remodeling. The effective healing of wounds is determined by both genetic and epigenetic alterations, because any changes in gene expression often lead to abnormal cellular behavior, which affects the production and proliferation, resulting in abnormal scarring or chronic wounds [103]. There are several genetic factors involved in wound healing that promote tissue repair and angiogenesis, such as Transforming growth factor‐1(TGF‐β), VEGF, and hypoxia‐inducible factor‐1 (HIF‐1) [104].

### 6.1 TGF‐β Signaling Pathway

In the TGF‐β signaling pathway, plasma membrane‐associated receptors like type 1 and type 2 positioned on the cell membrane are involved. Similarly, the ligands or signaling molecules like TGF‐β, NODAL, activin, BMPs, GDFs, and AMH bind to the receptors and start the pathway. As soon as the pathway begins, the intracellular signaling regulatory molecules (ISRM), like R‐Smad, further include their co‐types like Smad 1, 2, 3, 5, 8, 9, and co‐Smad like Smad 4, transfer signals from activated receptors positioned on the nuclear surface to control gene expression [105]. The type 1 receptor is also called the mediator of ISRM, and type 2 is the ligand or signaling molecule. In order to initiate the signaling pathway, ligands like TGF‐β bind to type 2 receptor monomers of TGIF present on the cell membrane, inducing the dimerization of monomers. Therefore, it attracts the type 1 monomers closely to type 2 receptors, which then initiates and exhibits kinase activity in type 1 receptors. This proposes that type 1 receptors have serine residues, which leads to the formation of a heterotetrameric complex of TGF‐β receptors, along with the activated type 1 receptor [106]. A similar mechanism is followed by the BMP signaling molecules, where the mediators for the intracellular signal vary. The signaling molecules, producing signals by TGF‐β, activin, NODAL, and GDFs, mediate signals through Smad 2 and Smad 3, whereas signaling through AMH, BMP, and some GDFs mediates signals through Smad 1, Smad 5, and Smad 8 or 9. Moreover, Smad 4 co‐4 acts as a common mediator that connects all the mediators.

**Figure 5:**
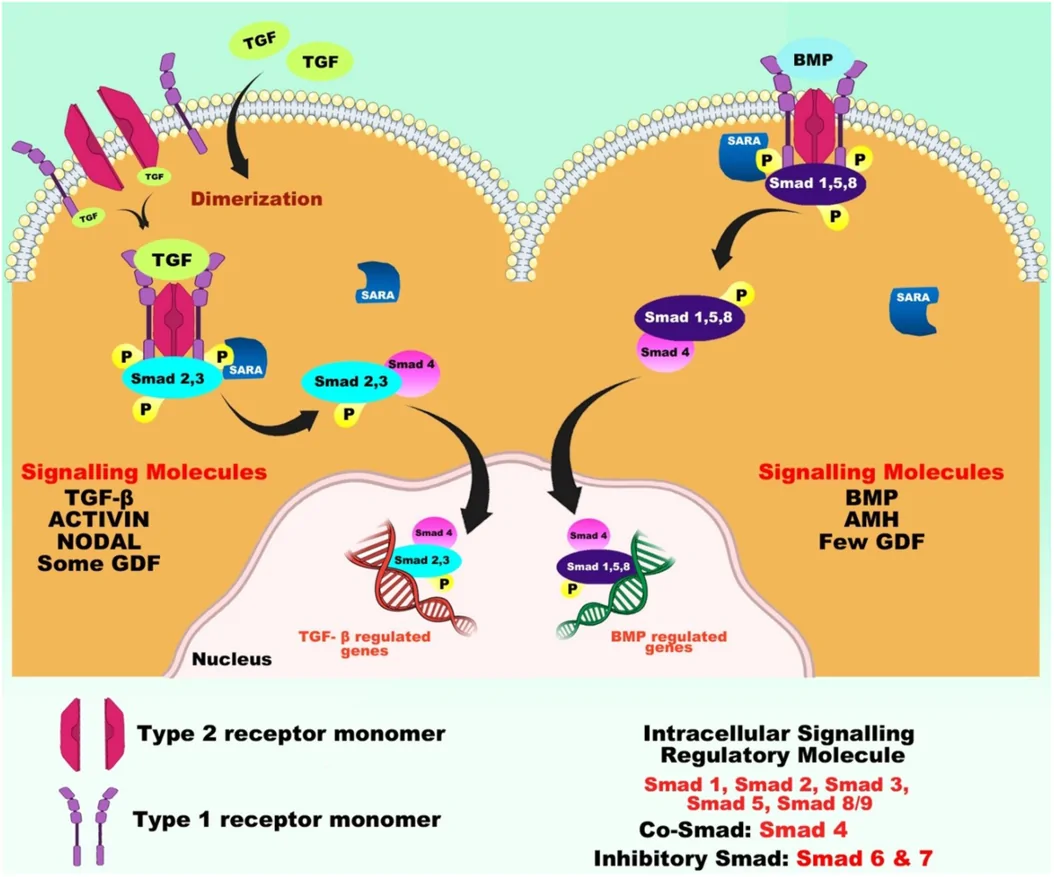
Outline of the TGF‐β and BMP signaling pathways, their Smad‐mediated transcriptional regulation.

Type 1 receptors recruit Smad 2 and 3, along with proteins like SARA, which allow the binding of Smad to the ELF‐rich region, which assembles the signaling complex on the type 1 receptor. The activated Smad 2 and 3 attract the co‐Smad 4, which forms a complex and interacts with signaling molecules. On the contrary, in the BMP‐initiated pathway, Smad 1, 5, and 8 bind along with SARA to the type 1 receptor and are phosphorylated like Smad 2 and 3, then bind with co‐Smad 4. Later, both molecules bind to specific promoters and receptors in the nucleus, leading to the activation of the transcription process, which binds specifically with the ISRM. Hence, the BMP molecules induce transcription of mRNAs involved in osteogenesis, neurogenesis, and other specification, whereas TGF‐β induces the transcription of mRNAs involved in apoptosis, ECM deposition [107], and immunosuppression. Figure 5 shows TGF‐signaling pathways, describing the receptor activation, transcriptional regulation, and Smad phosphorylation.

### 6.2 VEGF Signaling Pathway

The VEGF pathway involves ligands, or signaling molecules, such as VEGF A, VEGF B, VEGF C, and VEGF D. The receptors involved in this pathway are VEGF R1, VEGF R2, and VEGF R3, which are ultimately known as RTKs and belong to class 4 RTKs. The receptors are in two forms: inactive and active. The inactive form of receptors is the one in a monomeric form, where VEGF monomers are present, whereas the active form of receptors is the one in a dimeric form, where dimers of VEGF are present [71]. Due to cross‐phosphorylation by the ligand, it binds to receptors, exhibiting binding. The monomers of the VEGF receptors are positioned on the surface of the cell membrane, and the VEGF signaling molecules enter and bind to the monomeric receptors and initiate dimerization. This dimerization leads to the cross‐phosphorylation of tyrosine kinases, which activates the VEGF receptor. This receptor attracts PLC‐γ, which gets activated and attracts the PIP2 present on the cell membrane, which converts it into diacylglycerol (DAG), positioned on the cell membrane, and IP3 positioned in the cytoplasm [108].

**Figure 6:**
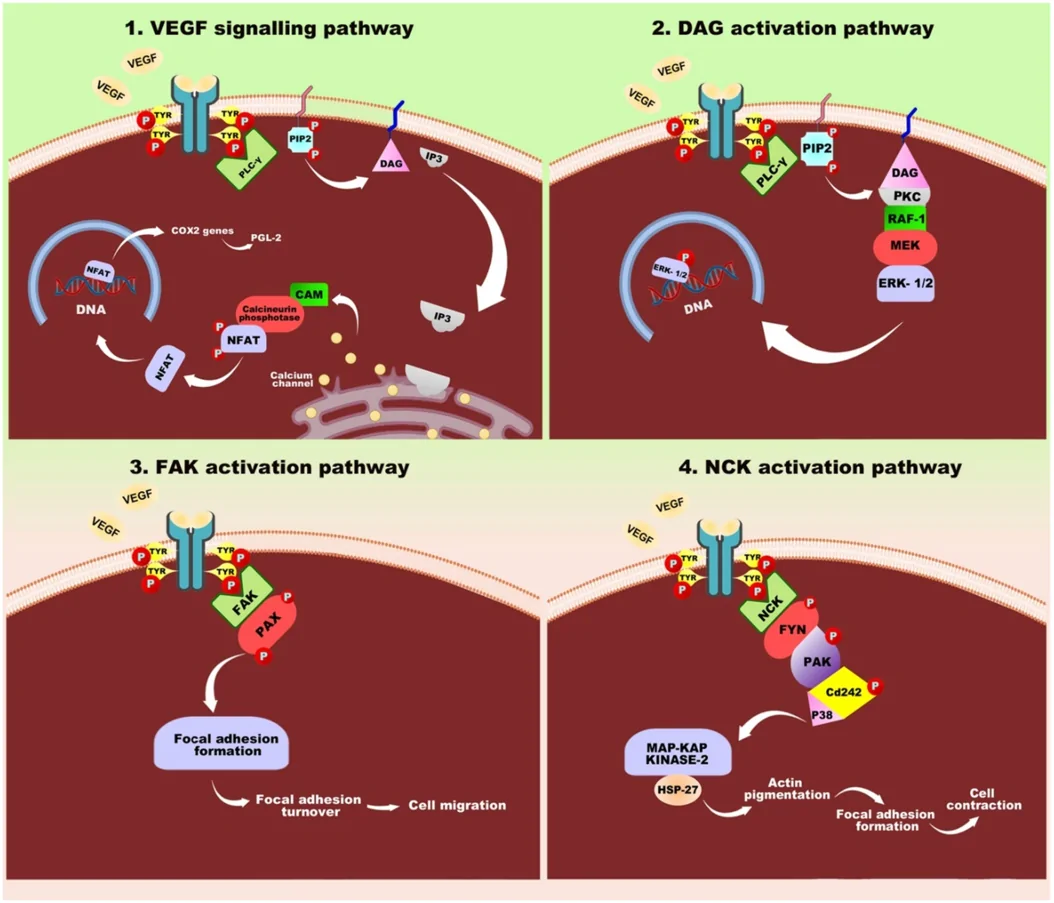
Crucial VEGF signaling pathways including PLCγ/IP_3_–Ca^2+^, DAG–PKC–ERK, FAK‐mediated adhesion turnover, and NCK–FYN–PAK–p38 coordinating endothelial activation, cytoskeletal remodeling, and migration.

During the IP3 pathway, the IP3 molecule enters the endoplasmic reticulum, opens the calcium channels, which release the calcium ions, and binds with the Calmodulin molecule to activate it. The activated Calmodulin molecule attracts the calcium ions, which are the phosphatase enzyme, and gets activated [109]. Later, these enzymes act on the phosphorylated NF80 molecule, which dephosphorylates it and enters the nucleus, where the transcription of cyclooxygenase‐2 (COX‐2) genes occurs. However, in addition to its expression, this COX‐2 gene is responsible for producing prostaglandin E2 (PGE2), which promotes angiogenesis by stabilizing VEGF expression and amplifying the downstream signaling cascades [110]. On the contrary, the DAG molecule activates the protein PKC, which is postulated to activate the protein redox factor‐1 (REF1), then activates the protein mitogen‐activated protein kinase (MEK). This protein further activates the transcription factor Extracellular signal‐regulated kinase 1 and 2 (ERK1/2), which enters the nucleus and initiates genes for proliferation. Similarly, the VEGF pathway activates the protein Focal adhesion kinase (FAK), which then activates the paired box proteins (PAX) and initiates focal adhesion formation, which ultimately leads to cell migration [111]. Figure 6 illustrates the EGF‐driven PLCγ–Ca^2+^, DAG–PKC–ERK, FAK, and NCK–PAK signaling pathways regulating endothelial activation and migration.

### 6.3 Effect of Mitochondrial Dysfunction on Genetic Regulation

Researchers stated that mitochondrial dysfunction not only affects oxidative stress but also affects several gene expressions involved in wound healing. Increased mitochondrial dysfunction changes the expression of transcription factors and signaling factors that manage the genes involved in promoting angiogenesis, proliferation, ECM deposition, and reducing inflammation. This dysfunction damages the VEGF‐signaling pathway by altering the production of endothelial cells, which carry essential nutrients and oxygen to the tissues, generating new ones. Similarly, the stubborn oxidative stress increases the signaling of the TGF‐β pathway, ultimately activates sustained myofibroblast production, and promotes excessive collagen deposition, ultimately leading to fibrotic or hypertrophic scarring. Thus, mitochondrial dysfunction not only alters wound healing by modifying cellular metabolism but also affects the expression of genes responsible for promoting rapid healing of wounds [112].

## 7 Epigenetic Mechanism in Wound Healing

Epigenetic mechanisms of wound healing require active and complex regulation of gene expression during each phase of the wound healing process. This includes a number of critical processes such as DNA methylation, histone modifications, and regulation of non‐coding RNA expression [113]. These processes coordinate the activation and expression of genes integral to cell division, movement, variation, and inflammation control for wound healing. Similarly, epigenetic modulation controls the overlapping phases of wound healing by changing the gene expression of repair genes [114].

### 7.1 DNA Methylation

DNA methylation, mainly 5‐methylcytosine placed by DNA methyltransferases (DMT), is removed by ten‐eleven translocation (TET)‐mediated oxidation. This approach gives a stable layer of transcriptional control, which is increasingly recognized as vital for fibroblast phenotype and scar persistence. Healed or fibrotic tissues produce fibroblasts that exhibit locus‐specific differentially methylated regions (DMRs) at the site of promoters and enhancers of genes. These genes encode proteins of the ECM, the TGF‐β signaling pathway, and contractile machinery. Genome‐wide methylome profiling of dermal and organ‐specific fibroblasts showed conserved and tissue‐specific methylation signatures associated with pathological fibrosis [115]. DNMT and TET activity can also be manipulated in vitro and in preclinical models to reverse fibrotic gene expression and reduce collagen deposition, showing their causal significance and therapeutic potential. Mechanistically, methylation changes act at a variety of regulatory levels: promoter CpG island methylation directly represses transcription; methylation at distal regulatory elements alters chromatin looping and enhancer activity, and affects cell‐state programs [116]. Age and environmental exposures (hypoxia, inflammation) further remodel the fibroblast methylome and may explain inter‐individual differences in scar severity and keloid propensity. Locus‐specific epigenome editing is a translational avenue under investigation, while circulating cell‐free DNA methylation signatures are being explored as a potential predictor of aberrant healing [117].

### 7.2 Histone Modifications

Post‐translational histone modifications such as acetylation, methylation, phosphorylation, and ubiquitination encode a fast and reversible chromatin language that controls gene expression required for functions such as hemostasis, inflammation, proliferation, and remodeling after injury [118]. H3K27ac and H3K4me3 are activating which are rapidly deposited at the promoters and enhancers of pro‐angiogenic and inflammatory genes in keratinocytes, endothelial cells, and immune populations in the early wound phase to permit rapid transcriptional induction [119]. Furthermore, resolution and remodeling have been associated with global reorganization of the chromatin landscape during wound healing, including erasure of activating histone acetylation marks and accumulation of repressive modifications such as H3K27me3 at genomic regions that need to become transcriptionally silenced to enable cellular differentiation and maturity of scar tissue. The contextual functions of histone‐modifying enzymes, namely histone acetyltransferases (HATs), histone deacetylases (HDACs), methyltransferases, and demethylases, modulate wound healing processes. Indeed, several studies have shown that aberrant HDAC activities in macrophages and EPCs contribute to impaired angiogenic signaling and sustained inflammation, leading to delayed healing of chronic wounds [120].

### 7.3 Non‐Coding RNAs

Non‐coding RNAs act as fast and reversible regulators of wound biology. They influence mRNA stability and translation, support chromatin regulators, and facilitate communication between cells through EVs [121]. MicroRNAs like miR‐126 and miR‐132 assist the growth of endothelium and thus blood vessel formation. In contrast, miR‐146a and similar types influence the way in which macrophage cells respond to inflammation. Changes in miRNA expression are a key feature of long‐standing, non‐healing wounds and fibrotic scars. Preclinical delivery of miRNA mimics or antagomirs, using nanoparticles, hydrogels, or engineered EVs, has shown the ability to modulate angiogenesis, reduce excessive inflammation, and lessen fibrosis in several wound models, in support of the concept of therapy feasibility [122]. Long non‐coding RNAs control wound transitions by recruiting chromatin modifiers to specific loci. They may act as ceRNAs that sequester miRNAs or directly impinge on transcription factor activity. Functional screens in human skin have identified lncRNAs, such as SNHG26 and STX17‐AS1. Circular RNAs, due to their covalently closed structure and stability, often function as miRNA sponges or protein interactors, and display injury‐responsive expression dynamics in skin. Initial studies implicate individual circRNAs in modulating fibroblast activation and endothelial behavior, and suggest their value as circulating biomarkers [123].

## 8 Pathological Mechanisms of Wound Healing

In wound healing, the pathological mechanism disrupts the normal repair mechanism, which leads to chronic wounds, abnormal scarring, and delayed tissue regeneration. These are due to the sustained effect of inflammation, disorganized cell activity, and broken molecular signals [124]. This mechanism involves various key factors in wound healing, including DNA methylation, histone modification, impaired cellular activation, and molecular dysfunctions. The pathology of scarred wounds is affected by long exposure to inflammation [125]. Hence, understanding these mechanisms leads to the intervention of new therapeutic strategies and promotes wound healing.

### 8.1 Impaired Healing: Chronic Wounds, Diabetic Ulcers, and Ischemic Wounds

Impaired healing is an extensive pathological form, where the usual healing process of the wound becomes altered and delayed for a longer period, ultimately leading to scarring. Though wound healing comprises highly efficient phases like hemostasis, inflammation, proliferation, and remodeling, complete healing of wounds will be difficult in cases where abnormal cell functions occur and healing factors are falsely activated, which ultimately leads to pathological scarring or chronic wounds [126]. Similarly, excluding chronic and pathological scarring, malnutrition also plays a major role because it alters the essential properties of wound healing, strength, inflammatory reaction, collagen, and fibroblast production [127].

Usually, in a normal healing scenario, the repair mechanisms flow smoothly via various stages. Initially, clotting forms, leading to partial fibrin, and produces signals to attract immune cells. This ultimately leads to the production of inflammation, which recruits neutrophils, macrophages, and fibroblast cells. These cells form a matrix bed, which initiates the formation of blood vessels, epithelial cells, and an ECM layer by providing tensile strength to the wounded site. Furthermore, the tissues undergo remodeling to produce a completely healed wound [128]. Unfortunately, the above process is disrupted in impaired healing because the healing process is often disturbed, leading to prolonged inflammation [129], where various enzymes are present at high levels, affecting tissue quality and minimal growth due to the presence of pathogens. Finally, these result in abnormal formation of tissues, limited movement of cells, and disruption of wound closure for a longer period.

### 8.2 Pathophysiology of Impaired Healing

#### 8.2.1 Prolonged Inflammation

A critical disadvantage of impaired healing is prolonged inflammation and chronic wounds. Rather than resolving in a few days, stubborn inflammation allows the non‐healing factors, like infections, biofilm, necrotic tissue, and recurrent trauma, to persist [130]. On the contrary, neutrophils initiate the secretion of enzymes like MMPs and elastase, which destroy the ECM [131] and growth factors, and cytokines. This alters the balance between MMPs and tissue inhibitors of metalloproteinases (TIMPs), which changes the activity of fibroblast production on the wound matrix [132]. Likewise, this promotes the inhibition of angiogenesis and cell movement, which prohibits entry into the proliferative phase.

#### 8.2.2 Diabetic Ulcers and Ischemic Wounds

Research states that about 2% of the residents living in urbanized countries suffer from diabetic ulcers, with an overall frequency of 6.3% [133]. The most crucial factors, like age, infection, wound type, oxygenation, and existing health issues, worsen the prolonged infection. On the contrary, the diabetic wound, or diabetic ulcer, sometimes known as a chronic wound, develops due to the effects of prolonged hyperglycemia, abnormal blood flow, neuropathy, and defective immune activity [134]. Similarly, the high level of glucose initiates the formation of advanced glycation end‐products (AGEs), which are the major culprits in damaging minor blood vessels, then activate the NF‐Kb signaling by connecting with RAGE receptors of endothelial cells [135]. This process either alters the functioning of endothelial cells or causes malfunctioning of the endothelial cells, resulting in hypoxia, which limits oxygen‐reliant processes like fibroblast generation, movement of keratinocytes, and collagen production. Likewise, the high level of ROS generation and mitochondrial impairment weakens ATP generation, leading to biological aging of cells [136]. Furthermore, peripheral nerve dysfunction removes the numbing feeling and alters the sensation of the foot, which causes the formation of ulcers due to sudden trauma, abnormal pressure circulation, and impaired autonomic regulation, leading to dry skin [137]. Hence, factors such as neurological, vascular, and metabolic factors together create a hypoxic, energy‐depleted wound bed incapable of organized tissue repair.

**Figure 7:**
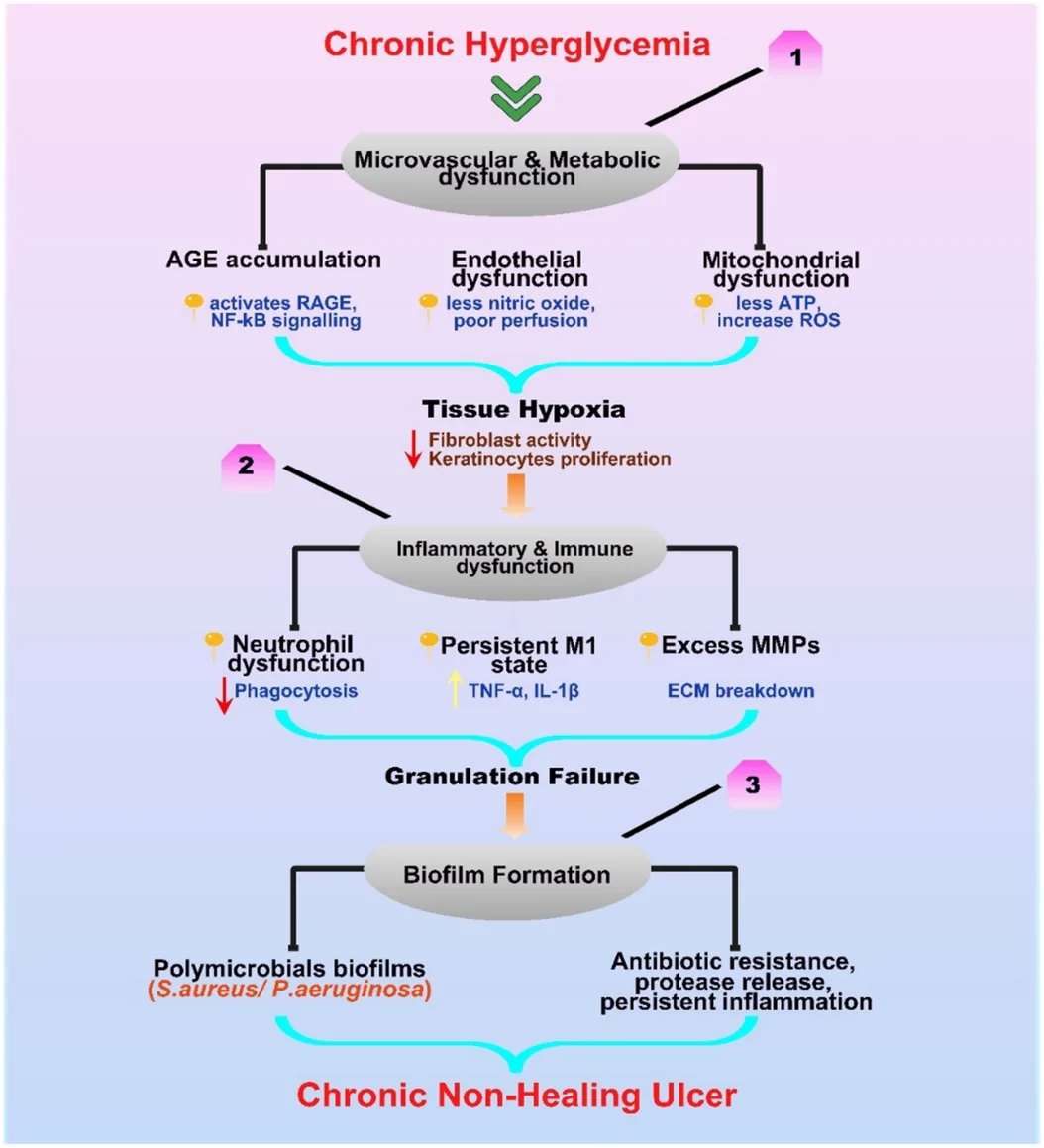
Illustrating how chronic hyperglycemia induces vascular and metabolic dysfunction, leading to tissue hypoxia, impaired immunity, excessive MMP activity, granulation failure, and biofilm‐mediated chronic wound ulcers.

Most importantly, ulcers occur due to these factors and the strong and persistent behavior of inflammation, ultimately leading to immunodeficiency. On the other hand, neutrophils display defective chemotaxis by releasing harmful chemicals like ROS and proteases, which damage the tissue [138]. Similarly, macrophages stay in the M1 phase and fail to make the transition to the essential M2 phase. This initiates the formation of MMP enzymes, which break the ECM of the tissue, and arrest the growth factors crucial for angiogenesis and tissue regeneration [139]. Unfortunately, pathogens like *Staphylococcus aureus* and *Pseudomonas aeruginosa* [140] sometimes worsen the condition by forming a biofilm layer, which resists the antibiotics' effect, ultimately leading to immune attack and producing enzymes by releasing toxins, which damage the new tissue growth and increase the inflammation for a prolonged period. Figure 7 represents the mechanism connecting chronic hyperglycemia to tissue hypoxia, immune abnormality, biofilm formation, and chronic or non‐healing wounds. Hence, these diabetic ulcers are more difficult to heal unless both the sugar level and biofilm or immune problems are addressed.

**Figure 8:**
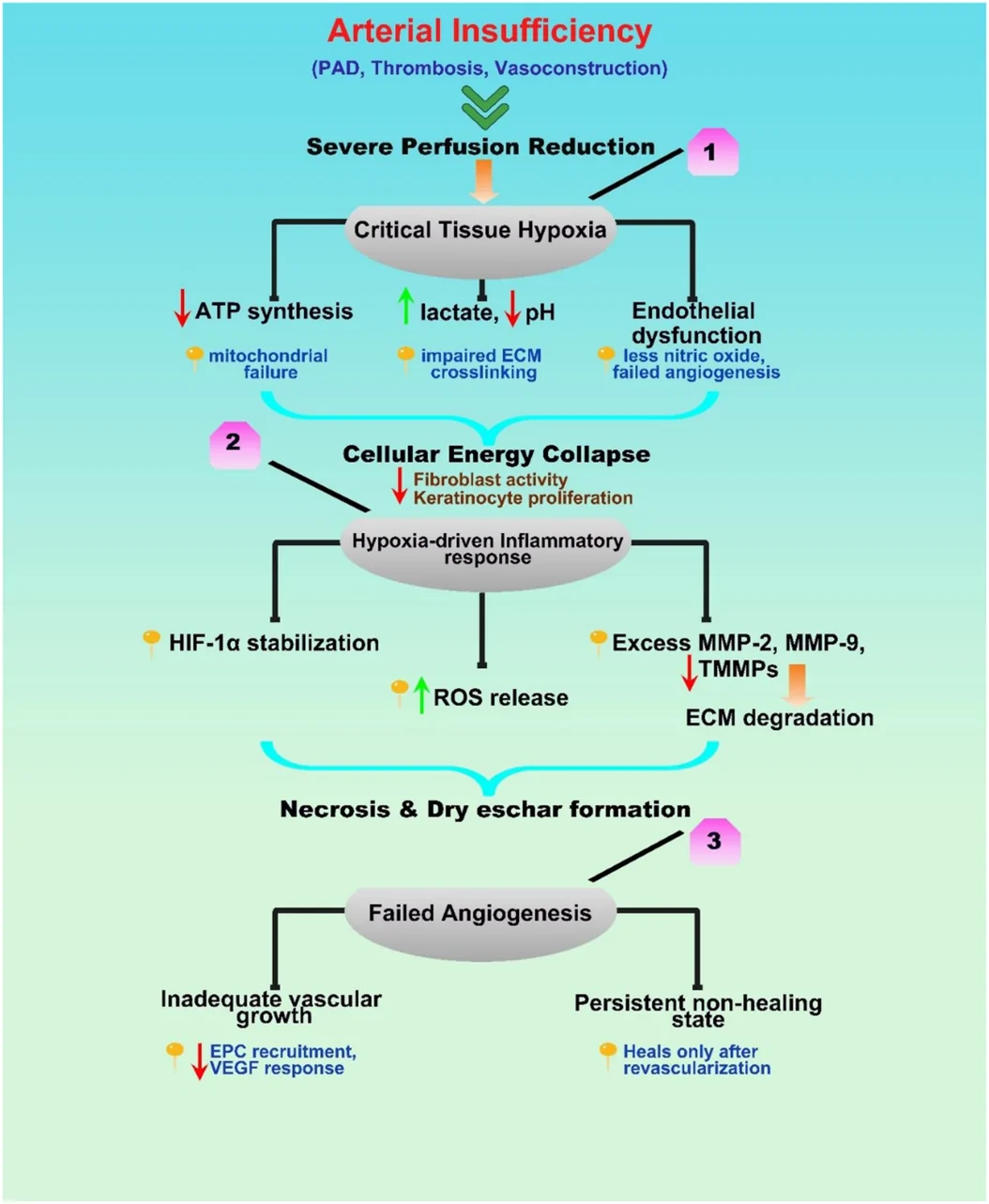
Mechanism of arterial insufficiency displaying how decreased perfusion causes hypoxia, cellular damage, ECM breakdown, poor angiogenesis, and chronic wounds.

Ischemic wounds are profoundly formed due to impaired arterial perfusion, which results in severe hypoxia and inhibits local cells from initiating metabolic signals [141]. The oxygen tension in these wounds often falls below the essential level for proliferation and collagen formation, where it then malforms due to the energy developed by ATP, and crucially depends on anaerobic glycolysis. Ultimately, this results in the accumulation of lactate, which creates an acidic microenvironment leading to the inhibition of enzymes or collagen crosslinking, and also weakens the connection of the ECM [142]. Followed by the disruption of the blood vessel lining, the production of nitric oxide was ultimately reduced, which made only a few cells form. Fortunately, this affects the growth of new blood vessel formation, and recurrent inhibition of blood vessels with a sudden increase in formation leads to the production of ROS, which damages the blood vessel lining, draws immune cells, and disrupts small blood vessels [143]. Ultimately, these wounds remain dry, necrotic, and poorly granulated unless perfusion is restored. Hypoxia, or low oxygen, actively prolongs inflammation and affects the tissue framework essential for healing [144]. The essential pathways, like HIF‐1α and NF‐kB, increase the inflammatory signals, which are crucial for attracting neutrophils. Poor, highly constricted blood vessels affect blood flow, which prevents their clearance, ultimately stopping macrophages from the M1 to M2 phase [145]. Consequently, the MMP enzymes, such as MMP‐2 and MMP‐9, break down elastin, collagen, and the basement membrane along with their natural inhibitors like TIMPs. However, arterial insufficiency disrupts the flow of blood, ultimately leading to hypoxia, inflammation, ECM breakdown, and less angiogenesis, as illustrated in Figure 8.

However, several conditions, such as an acidic environment, high levels of ROS production, and anaerobic bacteria, deteriorate the process, eventually leading to tissue death. The disruption and constriction of the blood supply interrupt and affect the stability of the ECM matrix, leading to abnormal or failed tissue regeneration [143]. It is crucial to design effective treatments for several pathogeneses of chronic wounds, which were described in Table 3.

**Table 3:** Mechanism‐driven therapeutic strategies for diabetic and ischemic wounds.

S. No	Pathogenic Association (diabetic/ischemic)	Mechanism of Impairment	Specific therapeutic strategy	Expected outcome	Reference
1.	**Diabetic:** Blood vessel dysfunction	Thick blood vessel lining Less NO production Low blood flow	Reduced heart workload NO contributors Platelet aggregation inhibitors Cutaneous oxygen therapies	Restores the growth of blood vessels, which helps to promote oxygen supply to tissues.	[146]
2.	**Diabetic:** Metabolic impairment	Degenerative progression High ROS production ATP depletion	Critical insulin or glucose monitoring ROS inhibitors Insulin sensitizer Mitochondrial supporting agents	Decreases metabolic stress, which enhances cellular activity and proliferation in tissues.	[147]
3.	**Diabetic:** Sustained Inflammation and an immune system disorder	Sustained M1 phase High protease synthesis	Topical immunomodulatory dressings Enzyme‐regulating biomaterials Multifunctional statin therapy M1/M2 macrophage modulation strategies	Enhances the healing process by reducing sustained inflammation.	[148]
4.	**Diabetic:** Biofilm formation	Severe resistance to antibiotics	Consistent tissue debridement Biofilm signal disruptors Silver nanoparticles Polyhexanide antiseptic Biofilm‐degrading enzymes	Increases the effects of antibiotics, which ultimately disrupts the biofilm formed.	[149]
5.	**Ischemic:** Advanced hypoxic state	Less oxygen supply Cell death	Direct wound oxygenation High‐pressure treatment Oxygen‐releasing biomaterials	It provides oxygen supply in the meantime, until new blood vessels form.	[150, 151]
6.	**Ischemic:** Arterial blockage	Peripheral arterial disorder Clotting Less blood vessel growth	Blood flow restoration procedures Clot‐preventing treatment Blood vessel‐inducing cytokines	It enhances the essential healing process, i.e., the blood supply.	[152, 153]
7.	**Ischemic:** Protease imbalance	Increased level of MMP production Decreased level of TIMPs	ECM‐promoting wound dressings MMPs inhibitors	It soothes ECM production, which protects the proliferative tissues.	[154, 155]
8.	**Ischemic:** Acidic tissue environment	Increased lactate level Less pH Abnormal collagen crosslinking	pH‐balancing wound dressings pH‐stabilizing hydrogel systems	ECM production can be increased by restoring the biochemical conditions.	[156, 157]
9.	**Ischemic:** Mitochondrial dysfunction	Increased hypoxia Increased ATP reduction	Nutrient‐driven cellular repair agents Tissue regeneration signaling therapy	It enhances the survival rate of cells until the blood flow is restored.	[136, 158]

### 8.3 Excessive Healing: Hypertrophic Scars, Keloids

Excessive healing involves the abnormal repair of tissues, where the normal repair mechanism is altered, which results in uncontrolled fibroblast and collagen production, and sustained ECM deposition [159]. Unfortunately, the wound gets trapped, repeatedly produces tissue, and releases inflammatory signals, which leads to the formation of scars, like hypertrophic and keloid scars [160]. These are clinically significant scars, which are formed due to alterations in essential healing characteristics like immune cells, fibroblasts, endothelial cells, and keratinocytes, along with their crucial signaling pathways like TGD‐βSmad, PI3K/Akt, MAPK, and Wnt/β‐catenin in the highly active phase [161]. However, the further consequences of these alterations are excessive collagen deposition, decreased apoptosis, sustained myofibroblast production, and reduced ECM remodeling, which increases the synthesis of MMPs and TIMPs. Hence, these lead to prolonged, non‐healing wounds or fibrotic scars, causing physical and psychological distress to the affected individuals [162].

#### 8.3.1 Hypertrophic Scars and Keloid Scars

Hypertrophic scars are formed due to sudden injury, trauma, surgical scars, or deep burn injuries. Sometimes they appear to be reddish colored, raised, and hard in nature, surrounded by the normal injured site. These scars appear for several weeks and flatten naturally [163]. Initially, the deposition of collagen III was higher, and then shifted to collagen I, which occurs due to the increased elevation in fibroblast production and collagen formation caused by the trigger of TGF‐β1 and TGF‐β2. Moreover, the stiffness and hardness of the scars are due to the differentiation caused by myofibroblasts [164]. These hypertrophic scars are clinically significant, with major symptoms like irritation, redness, and restricted movement, specifically on elbows, joints, chest, and shoulders [165].

On the contrary, keloid scars are more severe than hypertrophic scars, with growth beyond the normal wound limit, and sometimes take a prolonged time to heal. These scars are shinier, very hard, and sometimes appear like a lesion, with sharp pain at the wound site and in the surrounding area. Compared to hypertrophic scars, keloid scars take a long time to heal and persist for several months or even years. In particular, these scars were observed more often in people with dark skin, apparently a genetic condition. Alterations in specific HLA markers and in the genes that control TGF‐β signaling, p53 activity, and Smad transcription were highly involved. Basically, these scars are hyperactive and highly resistant to cell death. They produce a high level of collagen I, along with variations in growth factor synthesis, which increases vascular growth and mast cell infiltration, resulting in sustained inflammation [166]. This activates fibroblast production and ECM deposition, where significant surgical procedures or combined treatments are required to eliminate it. Moreover, the secreted fibroblast additionally produces epigenetic modifications, including alterations in DNA methylation patterns and histone acetylation patterns. As these patterns are responsible for controlling fibrotic gene expression even without external stimuli, alterations in them lead to abnormal gene expression, resulting in slow healing [167]. Finally, instead of a normal repair mechanism, the wound halts and is trapped in inflammation, with high fibroblast production, along with ECM deposition, caused by additional growth factor signals, imbalance of MMPs, and TMPs, leading to mechanical stress and fibroblast behavioral changes.

## 9 Immunological Mechanisms in Wound Healing

The wound healing process is a complicated, immune‐mediated process that involves innate and adaptive responses that coordinate to restore tissue integrity. At the site of the wound, early recruitment of neutrophils and macrophages begins inflammation; debris clearance continues by removing debris from the site; signaling processes are initiated that induce angiogenesis; and matrix remodeling occurs. Adaptive immune cells such as Tregs, Th17, and B cells balance resolution and fibrosis. In order to achieve healing, stromal, immune, and endothelial cells need to communicate via cytokines and metabolic cues.

### 9.1 Innate Immunity: Macrophage Polarization, Neutrophil Extracellular Traps (NETs)

A significant aspect of wound healing is the contribution of macrophages, which exhibit a remarkable degree of plasticity between pro‐inflammatory (M1‐like) and pro‐repair (M2‐like) phenotypes. In the early phase of wound healing, macrophages adopt an M1 profile driven by signals such as IFN‐γ, LPS, and TNF‐α, secreting pro‐inflammatory cytokines (IL‐1β, IL‐12, TNF) and reactive oxygen/nitrogen species to clear debris and pathogens [168]. This phase is required for initiating defense against immune cells, clearance of debris from the wound site, and recruitment of additional immune cells. Subsequently, a gradual transition to M2 macrophages is crucial, which is promoted by IL‐4, IL‐13, IL‐10, or efferocytosis of apoptotic neutrophils. M2 macrophages secrete anti‐inflammatory cytokines, growth factors, and promote angiogenesis, fibroblast activation, and ECM remodeling [169]. The failure of this transition, persistent M1 macrophages, or insufficient M2 induction has been associated with chronic inflammation, delayed wound closure, and scarring [170]. A significant body of recent research has identified epigenetics as a key regulator of this plasticity, including the acetylation and methylation of histones that affect polarization states in macrophages [171]. Moreover, macrophage metabolism plays a defining role; M1 macrophages depend on glycolysis and a broken TCA cycle, whereas M2 subtypes shift toward oxidative phosphorylation and Fatty‐acid oxidation, fully supporting reparative functions. In pathological settings such as diabetic wounds, macrophages display mitochondrial dysfunction, impaired efferocytosis of neutrophils, and failure to convert to the M2 phenotype, leading to sustained inflammation, poor angiogenesis, and increased fibrosis [172]. Emerging therapeutic strategies aim to modulate macrophage polarization to accelerate healing and mitigate fibrosis.

Neutrophils rapidly infiltrate injured tissue and take part in phagocytosis, degranulation, and the formation of NETs. NETs are a network of condensed chromatin combined with neutrophil elastase, myeloperoxidase, histones, and granular proteins. NET formation provides a robust mechanism to trap pathogens and limit infection spread, thereby contributing to early wound defense. Although they play an important role in wound repair, excessive or unresolved NET accumulation can prolong inflammation, damage surrounding tissue, impair re‐epithelialization, hinder angiogenesis, and promote fibrosis [37]. It has been shown that higher levels of circulating NET markers are correlated with poor outcomes in diabetic wounds as well as non‐healing ulcers [138]. The presence of NET components, such as histones or proteases, can activate fibroblasts, stimulate pro‐inflammatory macrophages, alter endothelial cell function, and promote the deposition of excessive extracellular matrix. Recent studies have shown that NETs may interact with other wound microenvironmental components (platelets, complement, and fibroblasts) and influence immunometabolic pathways [173]. Therapeutically, strategies such as DNase treatment, PAD4 inhibitors, nanoparticle‐based NET scavengers, or biomaterials that limit NET formation are under investigation to reduce NET‐driven delays in healing and fibrotic progression [37].

### 9.2 Adaptive Immunity: Tregs, Th17, B‐Cells in Repair and Fibrosis

Alongside innate immunity, adaptive immune subsets play an important role in wound healing, whether it leads to regeneration or fibrosis. Regulatory T cells (Tregs) have a major part in restraining excessive inflammation, supporting tissue regeneration, modulating fibroblast and endothelial responses, and suppressing pro‐fibrotic signals. Skin‐resident Tregs can reduce scars by limiting fibroblast activation and collagen deposition [174]. In contrast, T helper (Th17) cells secrete IL‐17A, IL‐22, and IL‐21, which promote neutrophil recruitment and inflammation and are also associated with dysregulated fibrotic outcomes [175]. An optimal Th17‐to‐Treg balance in the wound microenvironment is key to orderly repair; increased Th17 may predispose to persistent inflammation and fibrotic matrix deposition. B cells are less studied in wound repair, but emerging evidence indicates roles in antibody secretion, cytokine production, modulation of fibroblast behavior, and regulation of angiogenesis [170]. Treg deficiency, persistent Th17 activation, or B‐cell‐driven pro‐fibrotic cytokine production are examples of dysregulated adaptive responses with profound implications for hypertrophic scars, keloids, and non‐healing chronic wounds [176]. This rationale suggests adaptive immune modulation (e.g., promoting Treg expansion, reducing Th17 responses, optimizing B‐cell support) as one of the most promising therapeutic axes for directing healing towards regeneration and minimizing fibrosis [177].

### 9.3 Immunometabolism in Wound Healing

Immunometabolism controls immune cells to generate metabolic signals that control the phenotype and effector functions during the wound healing phase [178]. Following the injury, immune cells face a series of dysfunctions, such as an imbalance in oxygen tension, gradients in the nutrition level, and mediators of inflammation, accelerating dynamic shifts in metabolic pathways that directly dictate wound outcomes [179]. In the initial stage of the healing process, neutrophils and M1 macrophages depend entirely on aerobic glycolysis for high ATP production, ROS production, and cytokine secretion. This “Warburg‐like” metabolism generates intermediates such as succinate and fumarate, which stabilize H1F‐1α 1α and NF‐κB, reinforcing inflammatory gene expression and pathogen clearance [180]. In the microenvironment of the wounded site, a metabolic reprogramming begins with macrophage transitions toward oxidative phosphorylation (OXPHOS) and fatty‐acid oxidation (FAO), pathways that maintain the redox balance, support IL‐10 and TGF‐β secretion, and drive angiogenesis and matrix remodeling [181].

Mechanistically, this switch is activated by energy‐sensing molecules such as AMP‐activated protein kinase (AMPK) and mammalian target of rapamycin (mTOR). Hypoxic conditions or low nutrient presence activate AMPK, which promotes mitochondrial biogenesis and FAO, whereas mTOR hyperactivation sustains glycolytic, pro‐inflammatory states. Drug‐induced AMPK activation enhances macrophage M2 polarization and fibroblast alignment, accelerating wound healing. Similarly, sirtuin‐NAD+ pathways maintain mitochondrial integrity; depletion of NAD+ or SIRT1 impairs OXPHOS and perpetuates inflammation. Activation of cGAS‐STING and NLRP3 inflammasome signaling by mitochondrial stress links metabolic dysfunction to persistent fibrosis [182].

The wounded area suffers from metabolic constraints such as hypoxia, acidosis, and lactate accumulation, which stabilize HIF‐1α and inhibit histone deacetylases, coupling metabolism to epigenetic control of immune genes [183]. Metabolites such as itaconate and α‐ketoglutarate function as immunoregulatory signals: itaconate induces Nrf2‐driven antioxidant programs, while α‐ketoglutarate sustains demethylase activity, favoring anti‐inflammatory transcription. Cross‐talk between immune and stromal metabolism further refines repair, macrophage‐derived lactate promotes angiogenesis via endothelial HIF‐1α activation, whereas excessive glycolytic flux in fibroblasts amplifies collagen synthesis and scar formation [184].

## 10 Systemic Mechanisms

Systemic mechanisms determine the overall physiological and metabolic environment that regulates wound repair, inflammation, angiogenesis, and remodeling. When the body's overall balance is disrupted by chronic illness, poor nutrition, or aging, the immune system doesn't work as effectively, the skin takes longer to rebuild, and the production of ECM slows down [12]. All of this weakens the healing process, so understanding how these whole‐body factors work is essential for creating well‐rounded treatments that support proper wound repair.

### 10.1 Influence of Diabetes, Obesity, Malnutrition, and Aging

Diabetes mellitus is one of the most damaging systemic disorders that influences wound healing by means of constant hyperglycemia and oxidative stress, which in turn results in the formation of AGEs [185]. The accumulation of these products modifies structural and regulatory proteins, forming non‐enzymatic crosslinks that impair ECM remodeling and cellular adhesion. AGE binding to their receptors initiates downstream activation of the NF‐κB and JNK pathways, sustaining transcription of inflammatory mediators such as TNF‐α, IL‐6, and ICAM‐1. These signals maintain macrophages in an M1‐dominant phenotype and prevent the transition to a reparative M2 state. Inadequate VEGF‐driven angiogenesis is caused by disrupted H1F‐α stabilization produced by hyperglycemia during hypoxia. The activity of endothelial nitric oxide synthase (eNOS) is reduced as a result of impaired P13K/Akt signaling. Together, these molecular defects explain chronic inflammation, poor granulation tissue, and delayed epithelial closure in diabetic wounds [186].

Obesity differs from diabetes in wound healing because it exhibits parallel metabolic stress on the wound environment. Enlarged adipocytes release higher levels of pro‐inflammatory adipokines such as leptin, resistin, and TNF‐α, while at the same time, the release of adiponectin is reduced. This imbalance, hence, creates a pro‐inflammatory state that chronically activates the TLR4–NF‐κB pathway in macrophages and endothelial cells, driving systemic inflammation and slowing local tissue repair [187]. Excessive lipid accumulation leads to the formation of ROS through mitochondrial overload, which in turn activates p38 MAPK and inhibits collagen synthesis by fibroblasts. In addition, the chronic low‐grade inflammation and insulin resistance seen in obesity disturb the AMPK–mTOR balance, limiting the energy available for cell growth and migration.

Malnutrition, on the other hand, perturbs wound healing mainly by reducing the availability of amino acids and micronutrients required for cellular metabolism and matrix biosynthesis. Reduced essential amino acids limit proper collagen hydroxylation, and when vitamin C is lacking, prolyl hydroxylase cannot function effectively [188]. This combination leads to collagen fibrils that are weak and poorly matured. Trace minerals such as zinc and copper are crucial cofactors for DNA polymerases, and lysyl oxidase is also important because it supports keratinocyte growth and helps stabilize extracellular matrix proteins through crosslinking [189]. Low protein levels in the blood reduce plasma oncotic pressure, making it harder for nutrients to reach the wound site. This also extends the inflammatory phase, since the body cannot produce adequate amounts of complement proteins or immunoglobulins.

Inflammation is a long‐lasting, low‐grade form of inflammation that develops as age increases, largely because the body becomes less efficient in regenerating tissues [190]. As fibroblasts and keratinocytes age, they start to break down and respond less effectively to important growth factors such as TGF‐β1 and PDGF. This slows down the production of the extracellular matrix and the process of re‐epithelialization [191]. Aging also weakens mitochondrial function, leading to higher ROS levels, while declining NAD^+^ levels reduce sirtuin activity, affecting both DNA repair and metabolic control. In addition, age‐related stiffening of blood vessels reduces blood flow to tissues and slows the development of new blood vessels [192]. Altogether, these changes contribute to slower and often incomplete wound healing.

### 10.2 Cardiovascular and Renal Diseases Affecting Repair

Slowed wound healing is mostly linked with vascular system dysfunction. Cardiovascular issues like high blood pressure, hardened arteries, or abnormal cholesterol can damage blood vessel walls over time. This damage limits how much nitric oxide can be released, an important factor for keeping blood vessels flexible and preventing unwanted clots. As a result, new blood vessels have a harder time growing toward the wound, restricting the supply of oxygen and nutrients. The wound area ends up in a state that mimics low oxygen, which causes the body to activate factors like HIF‐1α. However, without follow‐up signaling through VEGF or eNOS, the formation of new blood vessels will be incomplete and function poorly. Alongside this, oxidative stress often increases, a chemical imbalance that hinders antioxidant enzymes such as superoxide dismutase (SOD) and catalase. This makes the cells trying to heal the wound more prone to damage and can really slow the recovery process [193].

Renal impairment, especially in chronic kidney disease (CKD), creates a widespread environment characterized by both the buildup of harmful toxins (uremia) and ongoing inflammation. Toxins like indoxyl sulfate and *p*‐cresyl sulfate accumulate and interfere with the movement of skin cells (keratinocytes) and slow down the growth of fibroblasts by triggering the AhR–NF‐κB signaling pathway [194]. In addition, anemia associated with kidney problems reduces tissue oxygen levels, and calcium and phosphate levels become imbalanced, leading to blood vessel calcification and making vessel walls less flexible. Constant inflammation, driven by molecules like IL‐6 and CRP, also disrupts the delicate balance between enzymes that break down the MMPs and their inhibitors, TIMPs, causing excessive breakdown of tissue support structures. When combined, heart and kidney problems change how the body manages metabolism and inflammation, resulting in wounds that remain low in oxygen, scarred, and less able to respond to normal healing signals [195].

### 10.3 Multi‐Organ Systemic Crosstalk

The neuroendocrine system plays a central regulatory role, largely through the hypothalamic‐pituitary‐adrenal (HPA) axis. During systemic stress, cortisol initially tempers excessive inflammation, but when cortisol remains high for too long, it slows fibroblast proliferation and weakens TGF‐β‐driven matrix formation. At the same time, the liver contributes through the acute‐phase response, producing fibrinogen, CRP, and serum amyloid A, all of which shape early inflammatory signaling and influence coagulation. Adipose tissue also participates through endocrine communication: leptin generally promotes macrophage activation and angiogenesis, while adiponectin acts as a counterbalance, dampening inflammation [196].

Growing evidence highlights the gut‐skin axis role in wound repair. Microbial metabolites and immune cell priming from the gut help regulate systemic immune tone, but when dysbiosis develops, cytokine patterns shift and regulatory T‐cell function weakens, pushing the body toward a more inflammatory state. Skeletal muscle adds yet another layer of communication by releasing myokines such as IL‐15 and irisin, which support angiogenesis and extracellular matrix remodeling by influencing fibroblast behavior. These interconnected systems create a coordinated biochemical environment that guides wound progression. When metabolic, endocrine, or vascular feedback loops become disrupted, the result is often prolonged inflammation, poor granulation, and ineffective tissue restoration [197].

## 11 Conclusion

The wound healing process is now recognized as a multi‐layered biological process rather than a normal, undeviating sequence. Cellular signaling pathways, molecular signaling pathways, immune modulation, control of epigenetic modifications, and systemic metabolic influences. Every phase, from clot formation to tissue regeneration, depends on cellular interactions, immune responses, and signaling pathways, including TGF‐β/Smad, PI3K/Akt, MAPK, and Wnt/β‐catenin. So any disturbances in these mechanisms result in impaired healing, as observed in diabetic ulcers and ischemic ulcers, and can lead to hypertrophic or keloid scars. The present clinical strategies often rely on wound appearance, overlooking the underlying mechanisms, which stresses the need for mechanistic re‐evaluation to improve diagnosis and guide targeted therapies. Normally, chronic wounds are not simply slow‐healing wounds. These wounds represent the unique biological features described by hypoxia, protease imbalance, altered immune responses, mitochondrial dysfunction, biofilm formation with antibiotic resistance, and epigenetic dysregulation. Likewise, abnormal healing or non‐healing wounds promote the unusual production of fibroblasts and prolonged collagen and inflammatory signals involving TGF‐β, MAPK, and oxidative stress, ultimately leading to sustained ECM deposition. As highlighted throughout this review, numerous mechanism‐based differentiations of wounds are critically required to provide personalized treatment strategies and bridge the gap between molecular pathology and clinical management. Hence, wound healing should be understood via the numerous systemic biological frameworks, interconnecting molecular signaling, mechanotransduction, immune metabolic responses, vascular growth, genetic and epigenetic modulation, and cellular plasticity.

## 12 Future Perspectives

Future research in wound healing must concentrate on symptom‐based care toward precision, mechanism‐driven medicine. Integrating multiple platforms, including proteomics, transcriptomics, genomics, epigenetics, and metabolomics, offers strong potential to identify diagnostic biomarkers that help predict and define patient‐specific wound‐healing strategies. Ultimately, this technique balances the challenges of treating similar wounds with different molecular mechanisms. Likewise, tools like single‐cell RNA sequencing, CRISPR‐mediated gene editing, spatial transcriptomics, and AI‐enabled wound monitoring enhance patient‐based treatment by using molecular profiles instead of surface appearances, improving the effectiveness of personalized wound medicine.

In contrast, nanotechnology and nanomaterials, environment‐adapting hydrogels, wound dressings, oxygen‐releasing scaffolds, ROS‐inhibiting nanoparticles, and biofilm‐disrupting agents can initiate the controlled production of growth factors, stem cell‐based exosomes, and microRNA mimics. Similarly, these materials can be modified and utilized to enhance specific functions, such as hypoxia, metabolic alteration, macrophage imbalance, and abnormal vascular growth. Finally, integrating bioinformatics, AI‐based healing models, wearable biosensors, and modified medicines may improve clinical support systems by enabling mechanistic, rather than trial‐and‐error, treatment. Hence, progress in wound care may come from identifying chronic wounds through molecular and other mechanisms, enabling targeted therapies and paving the way for functional, scar‐free wound healing.

## Author Contributions

**Devadass Jessy Mercy:** writing – original draft, methodology, data curation, formal analysis. **Koyeli Girigoswami:** formal analysis, investigation, data curation, writing – review and editing. **Agnishwar Girigoswami:** conceptualization, project administration, supervision, data curation, writing – review and editing.

## Funding

The authors have nothing to report.

## Ethics Statement

The authors have nothing to report.

## Conflicts of Interest

The authors declare no conflicts of interest.

## Data Availability

All data are available in the article.
